# Recombinant Mosquito Densovirus with *Bti* Toxins Significantly Improves Pathogenicity against *Aedes albopictus*

**DOI:** 10.3390/toxins14020147

**Published:** 2022-02-17

**Authors:** Khadija Batool, Intikhab Alam, Peiwen Liu, Zeng Shu, Siyu Zhao, Wenqiang Yang, Xiao Jie, Jinbao Gu, Xiao-Guang Chen

**Affiliations:** 1 Department of Pathogen Biology, Institute of Tropical Medicine, School of Public Health, Southern Medical University, Guangzhou 510515, China; khadijabatoolali@gmail.com (K.B.); peiwen20@foxmail.com (P.L.); zengshuu@163.com (Z.S.); zsy20170609@163.com (S.Z.); ywq920726@163.com (W.Y.); xiaojie51@yahoo.com (X.J.); gujinbao@smu.edu.cn (J.G.); 2 College of Life Sciences, South China Agricultural University, Guangzhou 510515, China; intikhabalam2013@gmail.com

**Keywords:** *Aedes aegypti* densovirus, *Bacillus thuringiensis*, toxicity, *Ae. albopictus*

## Abstract

Mosquito densoviruses (MDVs) are mosquito-specific viruses that are recommended as mosquito bio-control agents. The MDV *Aedes aegypti* densovirus (AeDNV) is a good candidate for controlling mosquitoes. However, the slow activity restricts their widespread use for vector control. In this study, we introduced the *Bacillus thuringiensis* (*Bti*) toxin Cry11Aa domain II loop α8 and Cyt1Aa loop β6-αE peptides into the AeDNV genome to improve its mosquitocidal efficiency; protein expression was confirmed using nanoscale liquid chromatography coupled to tandem mass spectrometry (nano LC-MS/MS). Recombinant plasmids were transfected into mosquito C6/36 cell lines, and the expression of specific peptides was detected through RT-PCR. A toxicity bioassay against the first instar *Aedes albopictus* larvae revealed that the pathogenic activity of recombinant AeDNV was significantly higher and faster than the wild-type (*wt*) viruses, and mortality increased in a dose-dependent manner. The recombinant viruses were genetically stable and displayed growth phenotype and virus proliferation ability, similar to wild-type AeDNV. Our novel results offer further insights by combining two mosquitocidal pathogens to improve viral toxicity for mosquito control.

## 1. Introduction

Mosquitoes are crucial vectors in the epidemiology of numerous human viral diseases, such as dengue, chikungunya, malaria, yellow fever, and the Zika virus disease, all of which provide significant health hazards as well as economic losses worldwide [1,2,3]. Vector bio-control strategies include the use of entomopathogenic bacteria, such as *Bacillus* spp. [4,5], and the use of mosquito densoviruses has also been considered [6,7]. In 1972, a Russian laboratory first identified a densovirus in *Ae. aegypti* larvae and named it the *Ae. aegypti* densovirus (AeDNV) [8]. Densoviruses (DNVs) are single-stranded, paraspherical DNA viruses (18–22 nm) that are non-enveloped and belong to the family Parvoviridae [7]. Mosquito densoviruses (MDVs) have been detected in many vector mosquito species, including *Aedes aegypti*, *Aedes albopictus*, *Anopheles gambiae*, *Anopheles sinensis*, *Culex pipiens*, and *Culex pipiens pallens* [9,10,11,12,13], that can replicate in numerous tissues, such as the midgut, anal papillae, Malpighian tubules, nerves, muscle fibers, fat body, and salivary glands, causing systemic infection [6,14]. Infected larvae have deformed segments and a cuticle of a semitransparent or whitish color with dense hypertrophied nuclei [15,16,17]. There are many limitations to wild-type MDV-based insecticides, leading to their restricted commercial use. The major disadvantage of MDVs is their slow activity, while single MDVs have different pathogenicity in different mosquito species. They work in a dose-dependent manner, depending on the viral titer and stage of infection [10,18]. In order to develop environmentally friendly mosquito control methods, researchers have evaluated microbial control strategies, mainly involving microbial genetic manipulation approaches. Paratransgenesis is the genetic manipulation of symbiotic microorganisms, including viruses, bacteria, and fungi [19,20,21,22]. Genetic approaches have been recommended as a promising tool for inhibiting the transmission of vector-borne pathogens by altering essential genes that function in vector reproduction, development, and host–pathogen interactions to prevent the development of vectors or pathogens [23,24,25,26]. Previously, we developed an *Ae. aegypti* recombinant densovirus (AeDNV) by inserting the insect-specific scorpion toxin gene *(BmK IT1)*, which improved the efficiency of the densovirus against *Ae. albopictus* [14]. These methods are more favorable due to simple manipulation, highly efficient gene transduction, the long-term persistence of gene expression, and the ability to produce long-lasting effects in vivo [27,28].

*Bacillus thuringiensis (Bt)* produces insecticidal crystal (Cry) and cytolytic (Cyt) proteins that exhibit high virulence to specific mosquito species [29,30,31]. Cry toxins have a wide range of insecticidal activity against Dipteran, Lepidopteran, and Coleopteran larvae, whereas Cyt toxins are mostly Dipteran-specific [32,33,34]. Interestingly, Cyt toxins are less pathogenic than Cry toxins, but they can enhance the activity of Cry toxins against mosquitos [34,35,36,37,38,39] and other insects [40,41,42,43,44,45]. The Cry toxin binds to putative receptors, including alkaline phosphatase (ALP), aminopeptidase (APN), and cadherin (CAD) receptors, in the midgut epithelium of mosquitoes [46,47]. Midgut proteases break down inactive Cry protoxins at specific sites, resulting in protease-resistant active fragments [30]. Cyt1Aa binds Cry11Aa through two exposed regions, including loop β6-αE (196-EIKVSAVKE-204) and part of β7. Cry11Aa, on the other hand, binds Cyt1Aa proteins via domain II-loops α8 and β4, which are also involved in the interaction of midgut receptors [43]. The specific regions of Cry11Aa and Cyt1Aa involved in binding interactions have been mapped [31]. Single-point mutations in Cry11Aa and Cyt1Aa reveal the key Cry11Aa (S259 and E266) and Cyt1Aa (K198, E204, and K225) residues involved in protein interaction and synergy [31]. Loop α8 in domain II of Cry11Aa is an important epitope that plays an essential part in interactions with different receptors [48]. A synthetic peptide with a sequence corresponding to loop α8 competes with the binding of brush-border membrane vesicles (BBMV) and site-directed mutations in loop α8 influence toxicity [48,49,50]. Genetically modified Cyt1Aa toxin (inserting loop-3 of the Cry1A toxin) revealed increased toxicity against Lepidopteran species, including *Menduca sexta* and *Plutella xylostella*, and showed synergism with Cry11Aa against third instar larvae of *Ae. aegypti* [44]. Both densoviruses and *Bti* are pathogenic to mosquitoes and have no toxic effect on unrelated species [51,52,53]. Studies on recombinant densoviruses with improved insecticidal efficacy are much needed, as limited literature is present. The objective of the present work was to assess the potential of recombinant densovirus as a biological control agent against the *Ae. albopictus* mosquito. The results provide a good theoretical basis for the development of potent larvicide applications of mosquito densovirus.

## 2. Results

### 2.1. Expression, Cloning, and Identification of Bti Toxins in AeDNV

**Figure 1:**
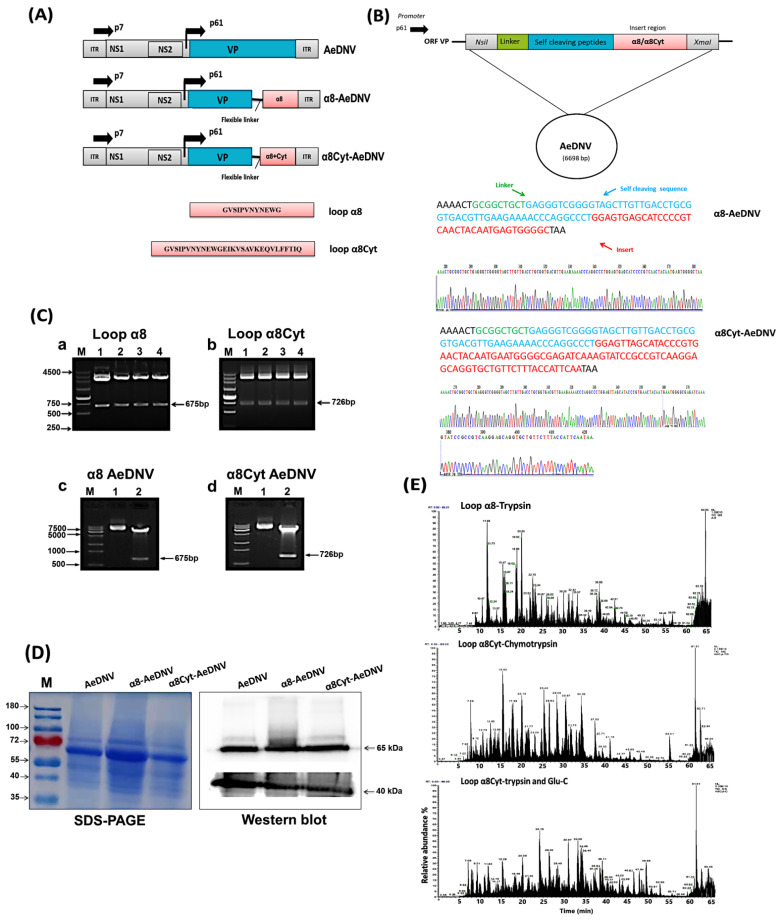
Plasmid design, cloning, and expression of *Bti* toxins in AeDNV. (**A**) Schematic presentation of recombinant *Ae. aegypti* densovirus (AeDNV) construct maps. The viral promoters p7 and p61 drive the expression of the *NS* and *VP* genes. (**B**) The specific DNA sequences of loop α8 and loop α8Cyt (red color) were inserted in VP (viral) proteins with a flexible linker (green color) and self-cleaving sequences (blue color). (**C**) Recombinant AeDNV with loop α8 and loop α8Cyt peptides were transformed. (**a**,**b**) The cloning of inserted fragment loop α8 and loop α8Cyt in cloning vector (PUC-57). Enzyme cutting showed a band at 675 bp and 726 bp sizes. (**c**,**d**) Transformation and enzyme digestion of insert loop α8 and loop α8Cyt in AeDNV plasmid. (**D**) Viral protein detection through SDS-PAGE and Western blot analysis. Total proteins were extracted from cell line C6/36 and showed a consistent band of 65 kDa NS1 protein and 40 kDa VP proteins in *wt* and recombinant viruses. Western blot analysis of NS and VP proteins through specific polyclonal antibodies detected viral proteins. (**E**) Representative total ion chromatograms (sum of all ion strength versus time) of loop α8 and loop α8Cyt peptides via nano LC-MS/MS spectrometry.

The nucleotide sequences of loop α8 (domain II) of the *Bti* toxin (Cry11Aa) gene and loop β6-αE of the Cyt1Aa toxin were cloned in a cloning vector (Figure 1B). Then, double enzyme digestion was carried out using *Xma-I/Nsi-I*, and the product sizes, i.e., 675 bp of loop α8 and 726 bp of α8Cyt, were confirmed (Figure 1C(a,b)). The fragments were transformed into the AeDNV viral genome and positive clones were selected after confirmation through enzyme digestion (Figure 1C(c,d)). Finally, the sequencing of the plasmids confirmed the successful insertion of the fragments without any frame shift (Sangon Biotech, Shanghai, China). Supercoiled plasmids were prepared, and 10 µg plasmid DNA was transfected into *Ae. albopictus* C6/36 cell lines. Five days post-transfection, total proteins were isolated, and we analyzed the viral proteins using SDS-PAGE. A band of 65 kDa NS1 proteins and a band of 40 kDa VP protein was observed in the total protein of recombinant and *wt*-AeDNV, (Figure 1D). Western blotting was performed to confirm the viral proteins in *wt* and recombinant viruses. The results revealed the presence of viral proteins in both *wt*-AeDNV and recombinant AeDNV viral proteins (Figure 1D). Nano LC-MS/MS analysis was carried out to further confirm the expression of the inserted peptides. The raw files collected via mass spectrometry were matched with the MaxQuant (1.6.2.10) database (www.maxquant.org accessed on 18 January 2022) and the proteins were identified (Table S1). The MS/MS spectrum of each peptide was recorded (Figure S2). In addition, total ion chromatograms showed the relative abundance of all ions in each sample (Figure 1E). These results revealed that the loop α8 and loop α8Cyt peptides were successfully detected with 100% sequence coverage (Figure S2).

### 2.2. Efficiency of Recombinant Virus in C6/36 Cell Lines and Insecticidal Efficacy against Ae. albopictus

**Figure 2:**
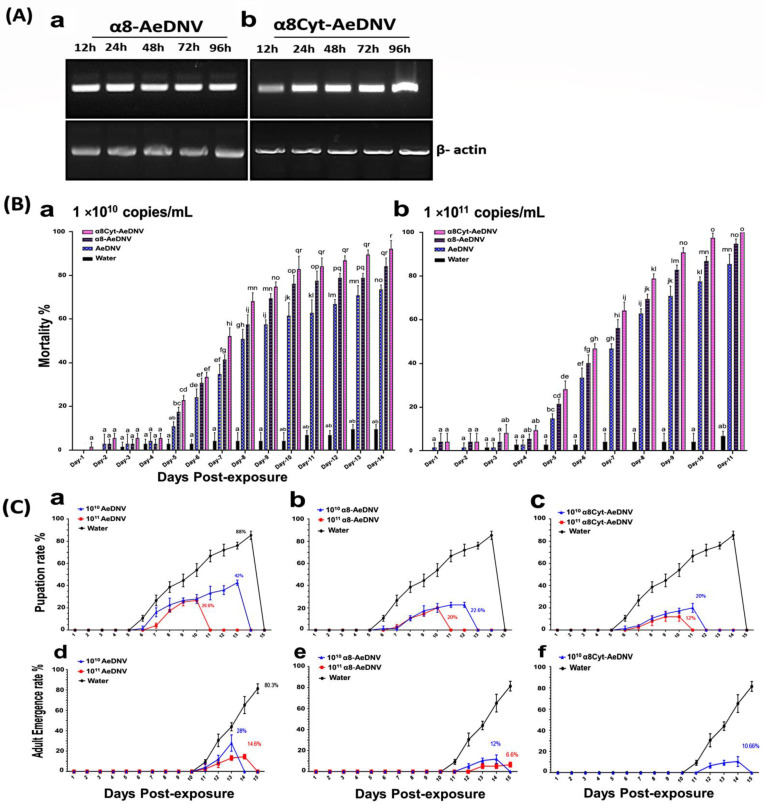
Efficiency of recombinant densovirus activity in RT-PCR and toxicity bioassay. (**A**) Detection of loop α8-AeDNV and loop α8Cyt-AeDNV fragment expression in C6/36 cell lines through RT-PCR. Loop α8 and loop α8Cyt messenger RNA (mRNA) accumulation at different time points (12, 24, 48, 72, and 96 h) were evaluated for (**a**) α8-AeDNV and (**b**) α8Cyt-AeDNV. (**B**) Toxicity bioassay analyses of recombinant and *wt*-AeDNV. The first instar larvae of *Ae. albopictus* were exposed to 1 × 10^10^ and 1 × 10^11^ copies/mL doses of densovirus. Bars indicate SDs from three biological replicates. The significance of the difference among the samples on different days was calculated by a one-way ANOVA followed by a Tukey HSD test using IBM SPSS ver. 22 (**B** (**a**,**b**)), and the significant difference is indicated by different letters (*p* < 0.05), while similar letters indicate no significant difference among the samples. (**C**) Rate of pupation (%) and emergence (%) of *wt* and recombinant densoviruses against *Ae. albopictus* larvae. (**a**) Rate of pupation in *wt*-AeDNV, (**b**) α8-AeDNV, and (**c**) α8Cyt-AeDNV. (**d**) Rate of emergence in *wt*-AeDNV, (**e**) α8-AeDNV, and (**f**) α8Cyt-AeDNV. Bars indicate SDs from three biological replicates.

The expression of the recombinant virus mRNA was determined using RT-PCR. Total RNA from the cells was isolated at several time points post-transfection. One-step RT-PCR was performed on 2 µg of total RNA with loop α8-AeDNV- and loop α8Cyt-AeDNV-specific primers. Product sizes for loop α8-AeDNV (255 bp) (Figure 2A(a)) and loop α8Cyt-AeDNV (275 bp) (Figure 2A(b)) were detected. In addition, the *Ae. albopictus β-actin* gene (GenBank accession # CB367652) was used as an internal control, with a 911 bp amplified product. The expressed fragments (loop α8-AeDNV and loop α8Cyt-AeDNV) were detected consistently 12 h to 96 h post-transfection in all samples, which revealed that the recombinant plasmids had high expressions of the inserted fragments at all time points post-transfection. 

To further explore the activity of the recombinant densoviruses, a toxicity bioassay was performed by infecting the first instar larvae of *Ae. albopictus* with 1 × 10^10^ copies/mL and 1 × 10^11^ copies/mL concentrations of recombinant and *wt* viruses under laboratory conditions. After 24 h post-exposure, larvae were washed and transferred to 200 mL of water in cups. Mortality rate was recorded daily. Recombinant viruses displayed an increase in cumulative mortality (Figure 2B(a,b)). The control samples showed 10–14% mortality. When larvae were treated with 1 × 10^10^ copies/mL recombinant and *wt* viruses, the mortality rate was significantly increased as compared to the *wt* virus, and the bioassay lasted for 14 days post-exposure. While when the viral concentration was raised to 1 × 10^11^ copies/mL, the mortality of the treatment groups with the recombinant virus was significantly higher than that of the *wt* virus at 11 days post-exposure. The larval stages were more vulnerable to the virus than the pupae or adults. The group of larvae treated with 1 × 10^10^ copies/mL of *wt*-AeDNV showed a 42% rate of pupation, while 28% pupae developed into adults. An increased concentration of 1 × 10^11^ copies/mL showed a 26.6% pupation rate, while 14.6% pupae developed into adults (Figure 2C(a,d)). The group of larvae treated with α8-AeDNV (1 × 10^10^ copies/mL) showed a 22.6% pupation rate, while 12% of pupae developed into adults. The 1 × 10^11^ copies/mL group showed a 20% pupation with 6.6% reaching adulthood (Figure 2C(b,e)). The larvae treated with α8Cyt-AeDNV at a concentration of 1 × 10^10^ copies/mL showed a 20% rate of pupation, while 10.66% developed into adult mosquitoes. Further, a treatment concentration of 1 × 10^11^ copies/mL resulted in decreased larvae survival with 12% pupation, and 100% of the larvae and pupae died on day 11 (Figure 2C(c,f)). These results suggest that the recombinant viruses are significantly lethal to larvae, more so than the *wt* virus. Two recombinants, including α8-AeDNV (containing loop α8) and α8Cyt-AeDNV (containing loop α8+loop β6-αE) displayed varied toxicity to some extent after several days post-exposure. Half lethal concentrations (LC_50_) of recombinant and *wt*-AeDNV were calculated through probit analysis in SPSS (version 22), and the LC_50_ of α8-AeDNV and α8Cyt-AeDNV was estimated to be 10^9.8^ copies/mL and 10^9.3^ copies/mL, respectively, while the LC_50_ of *wt*-AeDNV was 10^10.6^ copies/mL (Table 1). These results revealed that the recombinant viral activity was higher than *wt*-AeDNV. The half lethal time (LT_50_) was determined for recombinant viruses, including α8-AeDNV, α8Cyt-AeDNV, and *wt*-AeDNV, at concentrations of 1 × 10^10^ and 1 × 10^11^ copies/mL. Lethality time was reduced in recombinant virus-treated samples compared to that in AeDNV (Table 1). These findings suggest that recombinant viruses have the potential to improve viral toxicity.

### 2.3. Stability of the Recombinant Viruses

**Figure 3:**
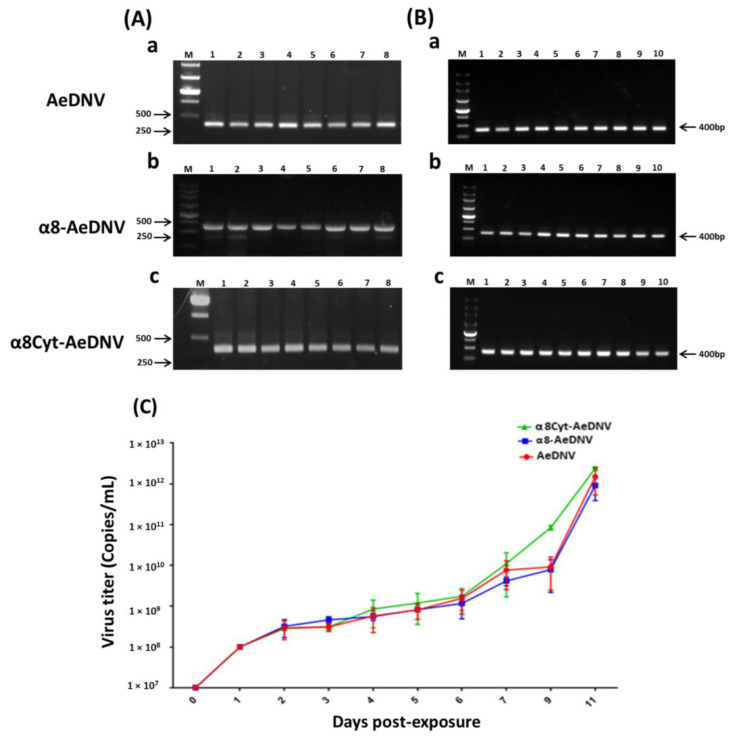
Genetic stability and proliferation analysis of recombinant virus. Virus genome stability was determined through genomic PCR of the infectious clones in *Stbl3* cells, with samples including (**A**-(**a**)) *wt*-AeDNV, (**A**-(**b**)) α8-AeDNV, and (**A**-(**c**)) α8Cyt-AeDNV. Genetic stability was tested by growing viruses serially in the C6/36 cell line, with samples including (**B**-(**a**)) *wt*-AeDNV, (**B**-(**b**)) α8-AeDNV, and (**B**-(**c**)) α8Cyt-AeDNV. (**C**) Recombinant virus proliferation activity in larval rearing water, measured with qPCR. Samples include *wt*-AeDNV (red color), α8-AeDNV (blue color), α8Cyt-AeDNV (green color). Bars indicate SDs from three biological replicates.

The genetic stability of α8-AeDNV, α8Cyt-AeDNV, and *wt*-AeDNV viruses was analyzed by serially growing recombinant virus strains (*E. coli stbl3*) in LB medium for 8 days (Figure 3A). C6/36 cells were infected with a virus at a concentration of 3 × 10^9^ copies/mL and grown for up to 10 serial passages (Figure 3B). Plasmid DNA and total RNA were extracted, and cDNA was used as a template for PCR to detect the complete amplicon of gene *NS1* (400 bp) on gel electrophoresis. We observed no sign of a mutation in the recombinants in LB medium (Figure 3A(b,c)) as compared to that in the *wt* virus strain (Figure 3A(a)). Furthermore, consistent results were found in C6/36 cell lines after gel electrophoresis, both in *wt*-AeDNV (Figure 3B(a)) and recombinant viruses (Figure 3B(b,c)) in up to 10 serial passages.

The proliferative ability of the recombinant viruses was further determined by infecting the first instar larvae of *Ae. albopictus* (200 larvae/cup) with 1 × 10^8^ copies/mL virus in 200 mL water in order to assess whether the infected larvae shed viral particles in the rearing water through horizontal transmission. Water samples were collected daily during larval growth and analyzed for viral accumulation using qPCR. The results showed an increase in viral concentration continuously from day 1 to day 11 post-exposure in all test samples (Figure 3C, Table 2). These results suggest that the recombinant viruses were genetically stable and had secondary transmission ability like that of the *wt* densovirus.

## 3. Discussion 

Recombinant MDVs that are used as a biological insecticide must retain their natural biological characteristics of vertical and horizontal transmission. Viruses with defective genomes lose their ability to reproduce and cause secondary transmission [27]. In order to retain the secondary transmission ability of densoviruses, we developed a non-defective densovirus in the present study by combining two mosquitocidal pathogens to increase efficiency, reduce resistance, and control the *Ae. albopictus* population. We constructed a recombinant densovirus (AeDNV) by introducing *Bti* Cry11Aa toxin domain II loop α8 and Cyt1Aa toxin loop β6-αE into the densovirus genome. The inserted peptides were highly expressed in *Ae. albopictus* cell lines and C6/36 cells at the transcription level. According to previous findings, all larval tissues (the midgut, nerves, muscle fibers, the Malpighian tubule, the foregut, and the hindgut) are possible sites of infection for AeDNV [14]. The inserted loops α8 and α8Cyt peptides of domain II (Cry11Aa) were linked with the *VP* gene through a flexible linker. The p7 and p61 viral promoters drive the expression of *NS* and *VP* genes (Figure 1A). When proteins are expressed, the specific loop fragments will bind to specific receptor sites through toxin receptor interactions and enhance the total toxicity [14,44]. The loop regions were selected on the basis of their toxin receptor binding interactions, which played a vital role in receptor interaction and toxicity. The cadherin receptor fragment containing CR7–11 (cadherin repeats 7–11) bound to Cry11Aa primarily through loop α8 (G257-Y268) of domain II toxin, whereas loop-3 of Cry11Aa was bound to CR11 (cadherin repeats) of *Ae*. *aegypti*. Further point mutation in α8 (E266A) led to the loss of toxin function to bind with the cadherin fragment that contains CR9 and CR10 repeats [49]. At least two Cry11Aa binding sites are found in the ALP receptor, with residue R59-G102 interacting with the exposed loop α8 from the Cry11Aa domain II, and residue N257-I296 interacting with Cry11Aa domain III [50]. The domain II region (V256-R360) was previously believed to be important for toxicity [54]. Mutations in the Cry11Aa synthetic peptides of domain II (loop α8, β4, and loop-3) were found to alter toxicity and receptor binding interaction [48]. Previously, the hybrid toxin of Cyt1A was modified by inserting the loop-3 fragment of domain II of Cry1Ab. The recombinant Cyt1A toxin was lethal to two agricultural pests (*M. sexta* and *P. xylostella*) and mosquito *Ae. aegypti* [44], even though the toxicity of wild-type Cyt1A is lower than that of the *Bti* Cry toxins [55,56,57]. Mutations in the Cry1A domain II loop regions, including loop-2, loop-α8, and loop-3, were found to be important in receptor interactions and toxicity in Lepidoptera [58,59,60].

Infectious cloning is a potent technique for genetically modified viral genomes. Unfortunately, full-length cloned viral DNA mutations have always been a major obstacle [61,62,63]. Virus inverted repeat (IR) sequences are mostly considered to be “hot spots” for genome instability. Plasmid instability is also caused by the *E. coli* host strains used in cloning procedures, e.g., the *TOP10* or *DH5α* strains. Variations might be possible due to duplications, mutations, and deletions during bacterial growth [64]. *Escherichia coli* S-strains have been developed to reduce some of these problems. Full-length cloned DNA is stable in S-strains; the S-strains are recommended for all cloning procedures of direct and inverted repeats, and are suitable for blue/white screening using vectors with complementation capabilities [27]. The genetic stability of both recombinant and *wt*-AeDNV in *E. coli Stbl3* cells was compared using a serial passage in LB medium and in cell line C6/36 cells. The results revealed a highly stable, viral infectious clone without any kind of mutation in the recombinant viruses as compared to that in the *wt* strain. Similarly, the viral *NS1* gene was consistently detected after being grown serially in C6/36 cells. The proliferative ability of densovirus was further determined by growing the virus in larval rearing water. The recombinant viruses replicated significantly in rearing water, similar to the replication of *wt*-AeDNV. The increase in viral concentration showed that the infected larvae released viral particles into the water environment, which facilitated the horizontal transmission of the virus [65]. The recombinant viral packaging is stable, allows for secondary virus transmission, and increases the viral titer in the water through infected larvae. 

A toxicity bioassay revealed a significant reduction in the treatment groups of larvae exposed to recombinant AeDNV compared to that in the *wt*-AeDNV group. Mortality increased with increasing viral concentration. The recombinant α8Cyt-AeDNV was more lethal to *Ae*. *albopictus* larvae than α8-AeDNV in higher concentrations. Previous experimental infection studies showed that AeDNV is more lethal to *Ae. albopictus* than *Ae. aegypti* [66]. Our results were consistent with a previous report in which maximum mortality occurred in the larval stage with AeDNV infection [67]. The enhanced toxicity of the recombinant densovirus toxin not only involves the strong binding interactions of the inserted fragments with the larval gut, but also the densovirus itself is a mosquito pathogen. It may also be due to some interaction between densovirus proteins and *Bti* toxin proteins. Unrelated proteins with different insect specificities can be combined to make potent insecticides. A previous report showed that Cyt1A can synergize *Bin* toxicity to otherwise resistant *Culex quinquefasciatus* and to refractory *Ae. aegypti* larvae, whose midgut cells lack *Binary (Bin)* toxin receptors [37]. Similarly, the Cry1Ab recombinant toxin, by inserting specific peptides into exposed loops of the domain II region of Cry1Ab, showed enhanced toxicity to a hemipteran insect *Nivalopavata lugens* [68]. More research is needed to study the virus–protein interaction strategies. The larvicidal potential of these novel recombinant densoviruses, as well as their bioactivity in other mosquito species, needs to be explored in the future. The dual approach could be useful for mosquito control, as well as for elucidating future vector control and management directions.

## 4. Materials and Methods

### 4.1. Mosquito Maintenance

The *Ae. albopictus* Foshan strain used in the current study was collected from the Guangdong Province, China, and maintained in the laboratory. Mosquitoes were raised at 28 °C with a 12/12 h light/dark photoperiod and 70–80% humidity. Larvae were reared in water containers and fed on turtle food (INCH-GOLD, Shenzhen, China) along with yeast powder in a 1:1 ratio. Cell lines of *Ae. albopictus* C6/36 were obtained from Professor Jingqiang Zhang’s laboratory, School of Life Science at Sun Yat-sen University, and cultured at 28 °C in 1640-RPMI medium that was supplemented with 10% fetal bovine serum (FBS).

### 4.2. Construction of Recombinant Plasmids

The *E. coli* strain *Stbl3* was used for the propagation of all plasmids. The insecticidal *Cry11Aa* gene (GenBank accession number M31737.1) domain II loop α8 (257-GVSIPVNYNEWG-268) and *Cyt1Aa* toxin gene (GenBank accession number AVA17337.1) loop β6-αE (196-EIKVSAVKEQVLFFTIQ-212) sequences were synthesized (Nanjing Genscript Biotech Co. Ltd., Nanjing, China). The sequences of the *Bti* toxins were fused to the VP1 gene of AeDNV, and its expression was controlled by the p61 promoter. Recombinant plasmids were further prepared and enzyme digestion *(Xma-I/Nsi-I)* was performed, followed by a transformation in the densovirus vector as described before [27]. We constructed two recombinants, with one recombinant virus having loop α8 and another having loop α8+loop β6-αE sequences. The inserted sequences were confirmed via DNA sequencing (Sangon Biotech, Shanghai, China). The pUCA vector is an infectious clone with the AeDNV genome (3981 bp) in pUC19, and was made available by Jonathan Carlson [69]. All constructs were confirmed by sequencing. The plasmids used in this study are shown in Figure 1A.

### 4.3. Cell line Transfection 

Cell line (C6/36) transfection was carried out according to a previously reported method [27]. Briefly, the monolayer of cells was grown to 85–90% confluence in Corning™ Costar™ Cell Culture Plates, (Thermo Scientific Life Technologies, Waltham, MA, USA), and fresh medium was provided one day prior to transfection. The medium was removed and the cells were washed with 200 μL ice-cold phosphate buffer (PBS: pH 7.2), thrice. Following the manufacturer’s protocol, C6/36 cell line transfection procedures were performed using a Lipofectamine-2000 (Thermo Scientific Life Technologies, Waltham, MA, USA). Supercoiled plasmids were extracted using the OMEGA Endo-Free Plasmid Midi Kit (Takara, Kyoto, Japan). The cells were transfected with 10 µg of plasmid DNA. The plates were incubated for 6 h at 28 °C, followed by washing with PBS (pH: 7.2), before adding 1640 RPMI medium supplemented with 5% FBS (Gibco BRL). The cells were collected at different time points post-transfection (12, 24, 48, 72, and 96 h) to detect the transcription and expression of Cry and Cyt fragments using RT-PCR. Total RNA was extracted using TRIzol reagent (Invitrogen, Carlsbad, CA, USA). One-step RT-PCR was performed using the EasyScript One-Step gDNA Removal and cDNA Synthesis SuperMix kits (Transgen-Biotech, Beijing, China). As an internal, endogenous control, the actin gene (Accession no. CB367652) was used (Table 3)

### 4.4. Production of Recombinant Virus

Recombinant and *wt* viruses were collected as indicated in a previous report [27]. In short, infectious plasmids were transfected in cell lines grown in T-25 cm^2^ Corning^®^ cell culture flasks. After incubating them for 5 days post-transfection, the cells were collected by cell scrapers, and then lysed by freezing and thawing three times, followed by centrifuging for 5 min, 1000× *g* at room temperature. The supernatants were collected and stored as *wt* and recombinant virus stocks.

### 4.5. Western Blotting and Nano LC-MS/MS Analysis

Cell lines C6/36 were transfected with 10 µg supercoiled plasmid DNA of the both recombinant and wild-type densoviruses. Five days post-transfection, total proteins were isolated using Pierce™ IP Lysis Buffer (Thermo Scientific Life Technologies, Waltham, MA, USA). Briefly, the cells were centrifuged at 1000× *g* for 5 min, after which the supernatants were discarded. Then, the cell pellet was washed with ice-cold PBS (pH: 7.2) and centrifuged again at 1000× *g* for 5 min, followed by the addition of ice-cold IP lysis buffer to the cell pellet (500 μL/50 mg cell pellet). Lysates were incubated on ice for 5 min with periodic mixing. After that, cell debris was removed by centrifugation for 10 min at ~13,000× *g* at 4 °C. Finally, supernatants were carefully collected in new tubes for downstream application. The total proteins were examined on 12% polyacrylamide gel (SDS-PAGE) with 120 V constant voltages. The quality of viral proteins was checked using the Western blot technique. Recombinant and *wt*-AeDNV proteins were separated on 10% SDS-PAGE and transferred to polyvinylidene difluoride (PVDF) membranes (0.2 μm) (Thermo Scientific, Shanghai, China). The membranes were blocked with 5% BSA in TBST (TBS buffer + 0.1% Tween-20) buffer for 2 h, followed by rinsing with TBST buffer. The membranes were further incubated with primary antibodies (rabbit anti-VP and rabbit anti-NS polyclonal antibodies, 1:1000) at 4 °C, overnight. The washing steps were repeated thrice; membranes were then incubated with a secondary antibody (goat anti-rabbit, 1:3000 dilutions). Finally, the results were observed using a color-developing reaction from the ECL Color Development Kit (Beyotime Biotech, Shanghai, China). 

The inserted peptide fragments were sequenced using the nano LC-MS/MS method (Biotech-Pack Scientific, Beijing, China). 

The reagents used in the method, including DL-dithiothreitol (DTT), formic acid (FA), methanol, iodoacetamide (IAA), and acetonitrile (ACN), were obtained from Sigma (St. Louis, MO, USA). Trypsin, Chymotrypsin, and Glu-C were bought from Promega (Madison, WI, USA). Ultrapure water was produced by a Millipore purification system (Billerica, MA, USA). We processed the samples using an in-solution digestion method. After reducing them to 10 mM DTT at 56 °C for 1 h and alkylating them with 20 mM IAA at room temperature in the dark for 1h, the enzyme was added into the protein solution separately at a ratio of 1:50, and the solution was incubated at 37 °C overnight. After digestion, a self-priming desalting column was used to desalt the peptides, and the solvent was evaporated in a vacuum centrifuge at 45 °C. The peptides were further dissolved in a solution containing 2% acetonitrile and 0.1% formic acid, vortexed well, and centrifuged at 13,200 rpm at 4 °C for 10 min. The supernatant was finally transferred to the sample EP tube for mass spectrometry analysis. 

The peptide mixtures were auto-sampled directly and retained on a C18-reversed phase column (15 cm length, 150 μm i.d.) packed with ReproSil-Pur C18-AQ resin (1.9 μm, 100 Å, Dr. Maisch GmbH, Ammerbuch, Germany) with the nanoLC Utimate 3000 system. The sample was separated within a 60 min linear gradient at a flow rate of 600 mL/min. Mobile phase A (99.5% water and 0.5% formic acid) and mobile phase B (80% acetonitrile and 20% 0.1% formic acid in water) were used, with an elution gradient: from 4% to 10% B for 5 min, from 10% to 22% B for 80 min, from 22% to 40% B for 25 min, from 40% to 95% B for 5 min, and from 95% to 95% B for 5 min. The nanoLC was directly interfaced with the Q Exactive™ Hybrid Quadrupole-Orbitrap™ Mass Spectrometer (Thermo Fisher Scientific). The mass spectrometry parameters were adjusted: the capillary temperature was 270 °C, and the spray voltage was 2.2 kV. The MS precursor m/z range was 300.0–1800.0, and the MS resolution was 70,000 at 400 m/z. The parameters for MS/MS were as follows: the scanning range for product ions began at m/z 100; isolation width: 3.00; normalized coll. energy: 40.0; activation type: CID; minimum signal required: 1500.0; activation Q: 0.250; activation time: 30.000; default charge state: 6; MS/MS based on data: up to the top 20 most intense peptide ions from the Orbitrap preview scan. Using MaxQuant (www.maxquant.org accessed on 18 January 2022), the raw MS files were analyzed and searched against a target protein database based on the sample characteristics (1.6.2.10). The following parameters were set: the protein modifications were carbamidomethylation (C) (fixed), oxidation (M) (variable), and acetyl (Protein N-term) (variable); the enzyme specificity was trypsin; the maximum missed cleavages were 2; the precursor ion mass tolerance was 20 ppm; and the MS/MS tolerance was 20 ppm. For downstream protein identification analysis, only high-confidence, identified peptides were chosen.

### 4.6. Toxicity Bioassay

The virus titers (copies/mL) of recombinant and *wt* viruses were determined using real-time quantitative PCR (qPCR), as reported in previous studies [27,67]. Briefly, densovirus plasmid of a known concentration was used to construct a qPCR standard curve (10^9^–10^5^) via the serial dilution of 10-fold plasmid (supplementary Figure S1). The half lethal concentrations (LC_50_) of viruses against first instar *Ae*. *albopictus* larvae were determined after exposure to *wt* and recombinant viruses at different concentrations (1 × 10^7^ to 1 × 10^11^ copies/mL) and statistically evaluated using SPSS Probit Analysis (version 22). Moreover, half lethal time (LT_50_) was assessed from larvae infected with the recombinant and *wt* viruses at concentrations of 1 × 10^10^ and 1 × 10^11^ copies/mL, respectively. The toxicity bioassay was performed with first instar *Ae. albopictus* larvae (25 larvae/replicate) treated with the recombinant virus and *wt* virus, while the control group was not treated. In the treatment groups, larvae were exposed to the same concentration of recombinant or *wt* virus in a total volume of 10 mL. In this toxicity test, we selected two concentrations of viruses (1 × 10^10^ copies/mL and 1 × 10^11^ copies/mL) to evaluate cumulative mortality. The control group, without treatment, was exposed to culture medium (C6/36 cells). After 24 h post-exposure, larvae were removed from the treatments, washed with double distilled water thrice, and transferred into new cups containing distilled water (200 mL); the larvae were fed regularly. Larval mortality was recorded each day until all the larvae died or became adults. Each experiment was performed in triplicate.

### 4.7. Determination of Genetic Stability of the Virus

The infectious clones of recombinant viruses in the *E. coli* strain (*stbl3*) were analyzed for genome stability to confirm the non-defective recombinants that hold the complete genome, according to a previous report [27]. The infectious clones including the recombinant and *wt* plasmids were serially passaged 8 times through overnight incubation in LB medium supplemented with ampicillin (100 mg/ul). Plasmid DNA was extracted daily and a PCR was performed to check for the presence of the viral *NS1* gene (Table 4).

Genome stability was also assessed by growing the recombinant and *wt* viruses in C6/36 cells. Briefly, cell lines were infected with the virus (3 × 10^9^ copies/mL) and serially passaged up to 10 times. Each time, total RNA was extracted; cDNA was used as a template in the PCR reaction for the detection of the viral *NS1* gene. Additionally, densovirus proliferative ability was determined for both recombinant and *wt* viruses by infecting first instar larvae (200 larvae/cup) with 1 × 10^8^ copies/mL of virus stocks. Larval rearing water was sampled every day for 11 days. The total genomic DNA was isolated using a MiniBEST Viral RNA/DNA Extraction Kit Ver.5.0 (Takara, Japan). Furthermore, qPCR reactions were carried out in a volume of 20 µL (10 µL of SyberGreen, 0.4 µL of forward and reverse primers, 1 µL of template DNA, and 8.2 µL of RNAse free water). Each sample was replicated three times as per the following amplification program: pre-incubation at 95 °C for 10 min, followed by 40 cycles of 95 °C for 5 s, 55 °C for 10 s, and 72 °C for 15 s. The results were analyzed using Light Cycler 480 software (Roche, Basel, Switzerland).

### 4.8. Statistical Analysis

All the graphs were generated using GraphPad Prism 7 software. The error bars indicate SDs from three independent (biological) replicates. In the bioassay, half lethal concentrations and half lethal time were calculated by probit analysis using IBM SPSS version 22. The significance of the difference among the samples at different days was calculated by a one-way ANOVA followed by a Tukey HSD test using IBM SPSS version 22, and the significant difference is indicated by different letters (*p* < 0.05).

## Figures and Tables

**Table 1:** LC_50_ and LT_50_ of *Ae. albopictus* larvae exposed to a wild-type and recombinant AeDNV.

Sample	LC_50_ (Copies/mL)		LT_50_ (Days)			
	Copies/mL	95% CI	1 × 10^10^ Copies/mL, 95% CI		1 × 10^11^ Copies/mL, 95% CI	
AeDNV	10^10.6^	10^10.09^–10^11.2^	8.7 d	(8.14–9.08)	7.28 d	(6.55–7.98)
α8-AeDNV	10^9.8^	10^9.39^–10^10.5^	7.61 d	(7.26–7.94)	6.49 d	(5.48–7.48)
α8Cyt-AeDNV	10^9.3^	10^8.9^–10^9.93^	6.71 d	(5.48–7.87)	5.85 d	(4.61–6.96)

**Table 2:** Recombinant virus proliferation activity in larval rearing water.

Days	*wt*-AeDNV	α8-AeDNV	α8Cyt-AeDNV
1	1 × 10^8^	1 × 10^8^	1 × 10^8^
2	2.93 × 10^8^	3.21 × 10^8^	2.86 × 10^8^
3	3.12 × 10^8^	4.63 × 10^8^	3.1 × 10^8^
4	5.75 × 10^8^	5.56 × 10^8^	8.55 × 10^8^
5	8.15 × 10^8^	8.24 × 10^8^	1.21 × 10^9^
6	1.56 × 10^9^	1.18 × 10^9^	1.76 × 10^9^
7	7.73 × 10^9^	4.19 × 10^9^	1.1 × 10^10^
9	9.24 × 10^9^	7.93 × 10^9^	8.42 × 10^10^
11	1.49 × 10^12^	9.14 × 10^11^	2.41 × 10^12^

**Table 3:** Primers for Reverse transcription polymerase chain reaction (RT-PCR) used in the study.

Primer Name	Sequences (5′ to 3′)	Annealing Temperature and Cycles	Product Size (bp)	Program Application
Loop α8 F	TTAGCCCCACTCATTGTAGTTGAC	55 °C, 35	255 bp	RT-PCR
Loop α8 R	CGAATGAGTACAAAACTACACCTC			
Loop α8Cyt F	TTGACGGCGGATACTTTGAT		275 bp	
Loop α8Cyt R	CGAATGAGTACAAAACTACACCTC			
β-Actin F	CACCAGGGTGTGATGGTCGG		911 bp	
β-Actin R	CCACCGATCCAGACGGAGT			

**Table 4:** Primers used in the polymerase chain reaction (PCR) and real-time qPCR of AeDNV.

Primer Name	Sequences (5′ to 3′)	Annealing Temperature and Cycles	Program Application
AeDNV-qF	AACCGATAGAACGAACAC	55 °C, 40	Virus quantification
AeDNV-qR	TTGGAGGACGACTGATTA		
pMDV-F	AACTACCAGGAGCAGGAT	55 °C, 30	PCR-based viral genome detection
pMDV-R	TGTATGTGCGTTGTCTTC		

## Data Availability

The data sets that support the conclusions of this article are included in this article.

## Supplementary Materials

The following supporting information can be downloaded at: https://www.mdpi.com/article/10.3390/toxins14020147/s1, Figure S1: Standard curve for plasmid AeDNV. A standard curve was built by making serial 10-fold dilutions of a linear plasmid at known concentrations (109–105). The results were analyzed using Light Cycler 480 software (Roche, Basel, Switzerland). Figure S2: Representative total ion chromatograms and MS/MS spectrum of α8-AeDNV and α8Cyt-AeDNV. (A) Total ion chromatograms (sum of all ion strength versus time) of (a) loop α8 and (b,c) loop α8Cyt. (B) MS/MS spectrum of each peptide in loop α8 and loop α8Cyt after enzyme digestion. (a) Loop α8 was digested by trypsin, while (b,c) loop α8Cyt was digested by chymotrypsin and tripsin&Glu-C. Table S1: The raw data collected by mass spectrometry through the MaxQuant database and the obtained results of protein identification.

## References

[1] Cheng G., Liu Y., Wang P., Xiao X. (2016). Mosquito defense strategies against viral infection. Trends Parasitol..

[2] Wang G.-H., Gamez S., Raban R.R., Marshall J.M., Alphey L., Li M., Rasgon J.L., Akbari O.S. (2021). Combating mosquito-borne diseases using genetic control technologies. Nat. Commun..

[3] Ligsay A., Telle O., Paul R. (2021). Challenges to Mitigating the Urban Health Burden of Mosquito-Borne Diseases in the Face of Climate Change. Int. J. Environ. Res. Public Health.

[4] Metzger M.E. (2021). A legacy of mosquito control through wetland management: A tribute to William E. Walton and his contributions to science and entomology. Wetl. Ecol. Manag..

[5] Docile T., Figueiró R., Molina O., Gil-Azevedo L., Nessimian J. (2021). Effects of *Bacillus thuringiensis* var. israelensis on the Black Fly Communities (Diptera, Simuliidae) in Tropical Streams. Neotrop. Entomol..

[6] Carlson J., Suchman E., Buchatsky L. (2006). Densoviruses for control and genetic manipulation of mosquitoes. Adv. Virus Res..

[7] Johnson R.M., Rasgon J.L. (2018). Densonucleosis viruses (‘densoviruses’) for mosquito and pathogen control. Curr. Opin. Insect Sci..

[8] Lebedeva O., Zelenko A., Kuznetsova M. (1972). The detection of viral infection in larvae of *Aedes aegypti*. Mikrobiol. J..

[9] Zhai Y.-g., Lv X.-j., Sun X.-h., Fu S.-h., Fen Y., Tong S.-x., Wang Z.-x., Tang Q., Attoui H., Liang G.-d. (2008). Isolation and characterization of the full coding sequence of a novel densovirus from the mosquito Culex pipiens pallens. J. General. Virol..

[10] Li J., Dong Y., Sun Y., Lai Z., Zhao Y., Liu P., Gao Y., Chen X., Gu J. (2019). A novel densovirus isolated from the asian tiger mosquito displays varied pathogenicity depending on its host species. Front. Microbiol..

[11] Liu L., Shen Q., Li N., He Y., Han N., Wang X., Meng J., Peng Y., Pan M., Jin Y. (2020). Comparative viromes of Culicoides and mosquitoes reveal their consistency and diversity in viral profiles. Brief. Bioinfor..

[12] Johnson R. Anopheles Gambiae Densovirus as a Viral Vector for the Expression of Small RNAs and Transgenes. https://etda.libraries.psu.edu/catalog/18678ruj130.

[13] Parry R., James M.E., Asgari S. (2021). Uncovering the Worldwide Diversity and Evolution of the Virome of the Mosquitoes *Aedes aegypti* and *Aedes albopictus*. Microorganisms.

[14] Gu J.-B., Dong Y.-Q., Peng H.-J., Chen X.-G. (2010). A recombinant AeDNA containing the insect-specific toxin, BmK IT1, displayed an increasing pathogenicity on Aedes albopictus. Am. J. Trop. Med. Hyg..

[15] Perrin A., Gosselin-Grenet A.-S., Rossignol M., Ginibre C., Scheid B., Lagneau C., Chandre F., Baldet T., Ogliastro M., Bouyer J. (2020). Mosquito densoviruses: The revival of a biological control agent against urban Aedes vectors of arboviruses. bioRxiv.

[16] Perrin A., Gosselin-Grenet A.-S., Rossignol M., Ginibre C., Scheid B., Lagneau C., Chandre F., Baldet T., Ogliastro M., Bouyer J. (2020). Variation in the susceptibility of urban Aedes mosquitoes infected with a densovirus. Sci. Rep..

[17] Buchatsky L. (1989). Densonucleosis of bloodsucking mosquitoes. Dis. Aquat. Org..

[18] Hirunkanokpun S., Carlson J.O., Kittayapong P. (2008). Evaluation of mosquito densoviruses for controlling *Aedes aegypti* (Diptera: Culicidae): Variation in efficiency due to virus strain and geographic origin of mosquitoes. Am. J. Trop. Med. Hyg..

[19] Wilke A.B.B., Marrelli M.T. (2012). Genetic control of mosquitoes: Population suppression strategies. Rev. Instit. Med. Trop. Sao Paulo.

[20] Upadhyay A., Hadiya J., Gharde S. (2021). Biocontrol: An effective tool for agricultural insect pests management. J. Pharm. Innov..

[21] de Castro Poncio L., Dos Anjos F.A., de Oliveira D.A., Rebechi D., de Oliveira R.N., Chitolina R.F., Fermino M.L., Bernardes L.G., Guimarães D., Lemos P.A. (2021). Novel sterile insect technology program results in suppression of a field mosquito population and subsequently to reduced incidence of dengue. J. Infect. Dis..

[22] Bliman P.-A. (2021). A feedback control perspective on biological control of dengue vectors by Wolbachia infection. Eur. J. Control.

[23] Mysore K., Sun L., Hapairai L.K., Wang C.-W., Roethele J.B., Igiede J., Scheel M.P., Scheel N.D., Li P., Wei N. (2021). A Broad-Based Mosquito Yeast Interfering RNA Pesticide Targeting Rbfox1 Represses Notch Signaling and Kills Both Larvae and Adult Mosquitoes. Pathogens.

[24] Khalil S., Munawar K., Alahmed A.M., Mohammed A. (2021). RNAi-Mediated Screening of Selected Target Genes against Culex quinquefasciatus (Diptera: Culicidae). J. Med. Entomol..

[25] Whitten M.M. (2019). Novel RNAi delivery systems in the control of medical and veterinary pests. Curr. Opin. Insect Sci..

[26] Dong S., Dong Y., Simões M.L., Dimopoulos G. (2021). Mosquito transgenesis for malaria control. Trends Parasitol..

[27] Liu P., Li X., Gu J., Dong Y., Liu Y., Santhosh P., Chen X. (2016). Development of non-defective recombinant densovirus vectors for microRNA delivery in the invasive vector mosquito, *Aedes albopictus*. Sci. Rep..

[28] Xu T.-L., Sun Y.-W., Feng X.-Y., Zhou X.-N., Zheng B. (2021). Development of miRNA-Based Approaches to Explore the Interruption of Mosquito-Borne Disease Transmission. Front. Cell. Infect. Microbiol..

[29] Brühl C.A., Després L., Frör O., Patil C.D., Poulin B., Tetreau G., Allgeier S. (2020). Environmental and socioeconomic effects of mosquito control in Europe using the biocide *Bacillus thuringiensis* subsp. israelensis (Bti). Sci. Total Environ..

[30] Silva-Filha M., Romão T.P., Rezende T.M.T., Carvalho K.D.S., Gouveia de Menezes H.S., Alexandre do Nascimento N. (2021). Bacterial Toxins Active against Mosquitoes: Mode of Action and Resistance. Toxins.

[31] Pérez C., Fernandez L.E., Sun J., Folch J.L., Gill S.S., Soberón M., Bravo A. (2005). *Bacillus thuringiensis* subsp. israelensis Cyt1Aa synergizes Cry11Aa toxin by functioning as a membrane-bound receptor. Proc. Natl. Acad. Sci. USA.

[32] Pardo-Lopez L., Soberon M., Bravo A. (2013). *Bacillus thuringiensis* insecticidal three-domain Cry toxins: Mode of action, insect resistance and consequences for crop protection. FEMS Microbiol. Rev..

[33] Soberón M., López-Díaz J.A., Bravo A. (2013). Cyt toxins produced by *Bacillus thuringiensis*: A protein fold conserved in several pathogenic microorganisms. Peptides.

[34] Das S.K., Pradhan S.K., Samal K.C., Singh N.R. (2021). Structural, functional, and evolutionary analysis of Cry toxins of Bacillus thuringiensis: An in silico study. Egypt. J. Biol. Pest Control.

[35] Cantón P.E., Reyes E.Z., De Escudero I.R., Bravo A., Soberón M. (2011). Binding of *Bacillus thuringiensis* subsp. israelensis Cry4Ba to Cyt1Aa has an important role in synergism. Peptides.

[36] López-Molina S., do Nascimento N.A., Silva-Filha M.H.N.L., Guerrero A., Sánchez J., Pacheco S., Gill S.S., Soberón M., Bravo A. (2021). In vivo nanoscale analysis of the dynamic synergistic interaction of *Bacillus thuringiensis* Cry11Aa and Cyt1Aa toxins in *Aedes aegypti*. PLoS Pathog..

[37] Nascimento N.A., Torres-Quintero M.C., Molina S.L., Pacheco S., Romão T.P., Pereira-Neves A., Soberón M., Bravo A., Silva-Filha M.H.N.L. (2020). Functional *Bacillus thuringiensis* Cyt1Aa is necessary to synergize Lysinibacillus sphaericus binary toxin (Bin) against Bin-resistant and-refractory mosquito species. Appl. Environ. Microbiol..

[38] Wirth M.C., Walton W.E., Federici B.A. (2015). Evolution of resistance in Culex quinquefasciatus (Say) selected with a recombinant *Bacillus thuringiensis* strain-producing Cyt1Aa and Cry11Ba, and the binary toxin, Bin, from Lysinibacillus sphaericus. Appl. Environ. Microbiol..

[39] Valtierra-de-Luis D., Villanueva M., Lai L., Williams T., Caballero P. (2020). Potential of Cry10Aa and Cyt2Ba, Two Minority δ-endotoxins Produced by *Bacillus thuringiensis* ser. israelensis, for the Control of *Aedes aegypti* Larvae. Toxins.

[40] Monnerat R., Pereira E., Teles B., Martins E., Praça L., Queiroz P., Soberon M., Bravo A., Ramos F., Soares C.M. (2014). Synergistic activity of *Bacillus thuringiensis* toxins against Simulium spp. larvae. J. Invertebr. Pathol..

[41] Rao P., Goswami D., Rawal R. (2021). Cry toxins of *Bacillus thuringiensis*: A glimpse into the Pandora’s box for the strategic control of vector borne diseases. Environ. Sustain..

[42] Yang J., Quan Y., Sivaprasath P., Shabbir M.Z., Wang Z., Ferré J., He K. (2018). Insecticidal activity and synergistic combinations of ten different Bt toxins against Mythimna separata (Walker). Toxins.

[43] Chattopadhyay P., Banerjee G. (2018). Recent advancement on chemical arsenal of Bt toxin and its application in pest management system in agricultural field. 3 Biotech.

[44] Torres-Quintero M.-C., Gómez I., Pacheco S., Sánchez J., Flores H., Osuna J., Mendoza G., Soberón M., Bravo A. (2018). Engineering *Bacillus thuringiensis* Cyt1Aa toxin specificity from dipteran to lepidopteran toxicity. Sci. Rep..

[45] Kamatham S., Munagapati S., Manikanta K.N., Vulchi R., Chadipiralla K., Indla S.H., Allam U.S. (2021). Recent advances in engineering crop plants for resistance to insect pests. Egypt. J. Biol. Pest Control.

[46] Liu L., Li Z., Luo X., Zhang X., Chou S.-H., Wang J., He J. (2021). Which Is Stronger? A Continuing Battle between Cry Toxins and Insects. Front. Microbiol..

[47] Valtierra-de-Luis D., Villanueva M., Berry C., Caballero P. (2020). Potential for *Bacillus thuringiensis* and Other Bacterial Toxins as Biological Control Agents to Combat Dipteran Pests of Medical and Agronomic Importance. Toxins.

[48] Fernández L.E., Pérez C., Segovia L., Rodríguez M.H., Gill S.S., Bravo A., Soberón M. (2005). Cry11Aa toxin from *Bacillus thuringiensis* binds its receptor in *Aedes aegypti* mosquito larvae through loop α-8 of domain II. FEBS Lett..

[49] Chen J., Aimanova K.G., Fernandez L.E., Bravo A., Soberon M., Gill S.S. (2009). *Aedes aegypti* cadherin serves as a putative receptor of the Cry11Aa toxin from *Bacillus thuringiensis* subsp. israelensis. Biochem. J..

[50] Fernandez L.E., Martinez-Anaya C., Lira E., Chen J., Evans A., Hernández-Martínez S., Lanz-Mendoza H., Bravo A., Gill S.S., Soberón M. (2009). Cloning and epitope mapping of Cry11Aa-binding sites in the Cry11Aa-receptor alkaline phosphatase from *Aedes aegypti*. Biochemistry.

[51] El-Far M., Li Y., Fédière G., Abol-Ela S., Tijssen P. (2004). Lack of infection of vertebrate cells by the densovirus from the maize worm Mythimna loreyi (MlDNV). Virus Res..

[52] Empey M.A., Lefebvre-Raine M., Gutierrez-Villagomez J.M., Langlois V.S., Trudeau V.L. (2021). A Review of the Effects of the Biopesticides *Bacillus thuringiensis* Serotypes israelensis (Bti) and kurstaki (Btk) in Amphibians. Arch. Environ. Contam. Toxicol..

[53] Schweizer M., Miksch L., Köhler H.R., Triebskorn R. (2019). Does Bti (*Bacillus thuringiensis* var. israelensis) affect Rana temporaria tadpoles?. Ecotoxicol. Environ. Saf..

[54] Yamagiwa M., Sakagawa K., Sakai H. (2004). Functional analysis of two processed fragments of *Bacillus thuringiensis* Cry11A toxin. Biosci. Biotechnol. Biochem..

[55] van Frankenhuyzen K. (2013). Cross-order and cross-phylum activity of *Bacillus thuringiensis* pesticidal proteins. J. Invertebr. Pathol..

[56] Federici B.A., Bauer L.S. (1998). Cyt1Aa protein of *Bacillus thuringiensis* is toxic to the cottonwood leaf beetle, Chrysomela scripta, and suppresses high levels of resistance to Cry3Aa. Appl. Environ. Microbiol..

[57] Van Frankenhuyzen K., Tonon A. (2013). Activity of *Bacillus thuringiensis* cyt1Ba crystal protein against hymenopteran forest pests. J. Invertebr. Pathol..

[58] Rajamohan F., Alzate O., Cotrill J.A., Curtiss A., Dean D.H. (1996). Protein engineering of *Bacillus thuringiensis* δ-endotoxin: Mutations at domain II of CryIAb enhance receptor affinity and toxicity toward gypsy moth larvae. Proc. Natl. Acad. Sci. USA.

[59] Lee M.K., Jenkins J.L., You T.H., Curtiss A., Son J.J., Adang M.J., Dean D.H. (2001). Mutations at the arginine residues in α8 loop of *Bacillus thuringiensis* δ-endotoxin Cry1Ac affect toxicity and binding to Manduca sexta and Lymantria dispar aminopeptidase N. FEBS Lett..

[60] Rajamohan F., Hussain S.-R.A., Cotrill J.A., Gould F., Dean D.H. (1996). Mutations at domain II, loop 3, of *Bacillus thuringiensis* CryIAa and CryIAb δ-endotoxins suggest loop 3 is involved in initial binding to lepidopteran midguts. J. Biol. Chem..

[61] Blaney J.E., Durbin A.P., Murphy B.R., Whitehead S.S. (2006). Development of a live attenuated dengue virus vaccine using reverse genetics. Viral Immunol..

[62] Ruggli N., Rice C.M. (1999). Functional cDNA clones of the Flaviviridae: Strategies and applications. Adv. Virus Res..

[63] Choi V.W., Asokan A., Haberman R.A., Samulski R.J. (2007). Production of recombinant adeno-associated viral vectors for in vitro and in vivo use. Curr. Protoc. Molec. Biol..

[64] Hirsch M. (2015). Adeno-associated virus inverted terminal repeats stimulate gene editing. Gene Ther..

[65] Sun Y., Dong Y., Li J., Lai Z., Hao Y., Liu P., Chen X., Gu J. (2019). Development of large-scale mosquito densovirus production by in vivo methods. Parasites Vectors.

[66] Kittayapong P., Baisley K.J., O′Neill S.L. (2001). A mosquito densovirus infecting *Aedes aegypti* and Aedes albopictus from Thailand. Am. J. Trop. Med. Hyg..

[67] Ledermann J.P., Suchman E.L., Black W.C., Carlson J.O. (2004). Infection and Pathogenicity of the Mosquito Densoviruses AeDNV, HeDNV, and APeDNV in *Aedes aegypti* Mosquitoes (Diptera: Culicidae). J. Econ. Entomol..

[68] Shao E., Lin L., Chen C., Chen H., Zhuang H., Wu S., Sha L., Guan X., Huang Z. (2016). Loop replacements with gut-binding peptides in Cry1Ab domain II enhanced toxicity against the brown planthopper. Nilaparvata lugens (Stål). Sci. Rep..

[69] Wang S., Ghosh A.K., Bongio N., Stebbings K.A., Lampe D.J., Jacobs-Lorena M. (2012). Fighting malaria with engineered symbiotic bacteria from vector mosquitoes. Proc. Natl. Acad. Sci. USA.

