# Supplementary material for: Recombinant Mosquito Densovirus with Bti Toxins Significantly Improves Pathogenicity against Aedes albopictus
Source: Toxins (Basel). 2022 Feb 17;14(2):147. doi: 10.3390/toxins14020147 (PMC8879223; doi:10.3390/toxins14020147)
Supplement: Supplementary file 1 [file toxins-14-00147-s001.zip › toxins-1582539-supplementary.pdf]

# Supplementary Materials: Recombinant Mosquito Densovirus with *Bti* Toxins Significantly Improves Pathogenicity against *Aedes albopictus*

**Khadija Batool, Intikhab Alam, Peiwen Liu, Zeng Shu, Siyu Zhao, Wenqiang Yang, Xiao Jie, Jinbao Gu and Xiao-Guang Chen**

| | |
|---|---|
| Slope | -3.648 |
| Efficiency | 1.88 |
| Error | 0.22 |
| R^2 | 1 |
| Y-Intercept | 43.1 |

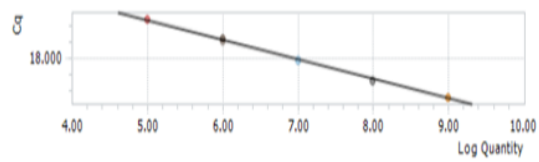


**Figure S1.** Standard curve for plasmid AeDNV. A standard curve was built by making serial 10-fold dilutions of a linear plasmid at known concentrations (109-105). The results were analyzed using Light Cycler 480 software (Roche, France).

**Peptide analysis of inserted loops through nano LC-MS/MS.**

**(A) a. $\alpha$8-trypsin**

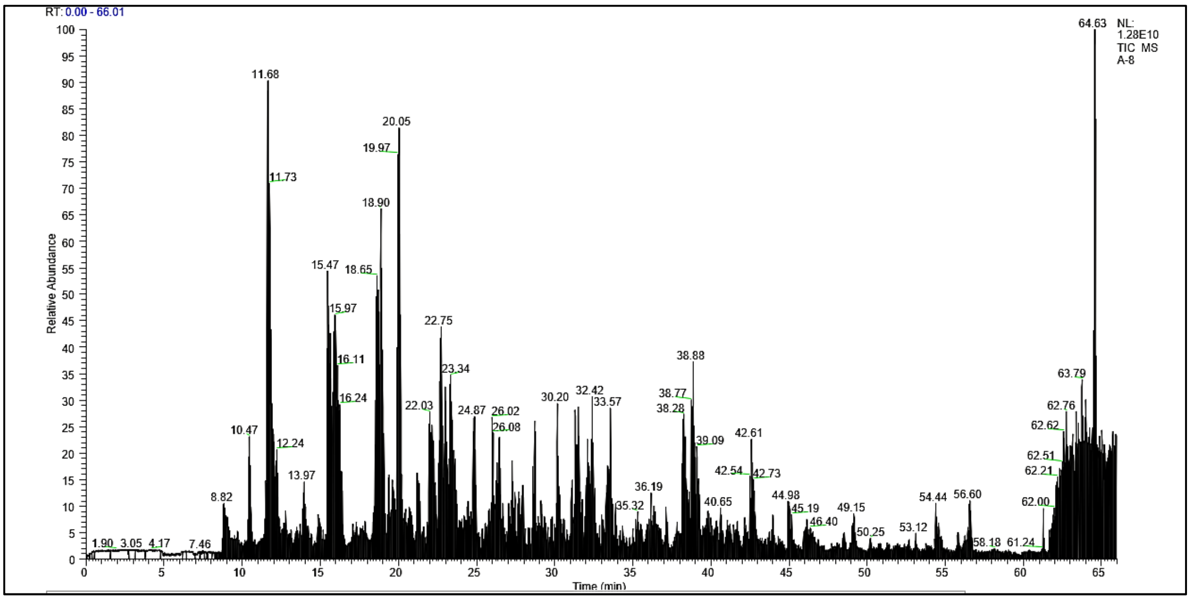


**b. $\alpha$8Cyt-Chymotrypsin**

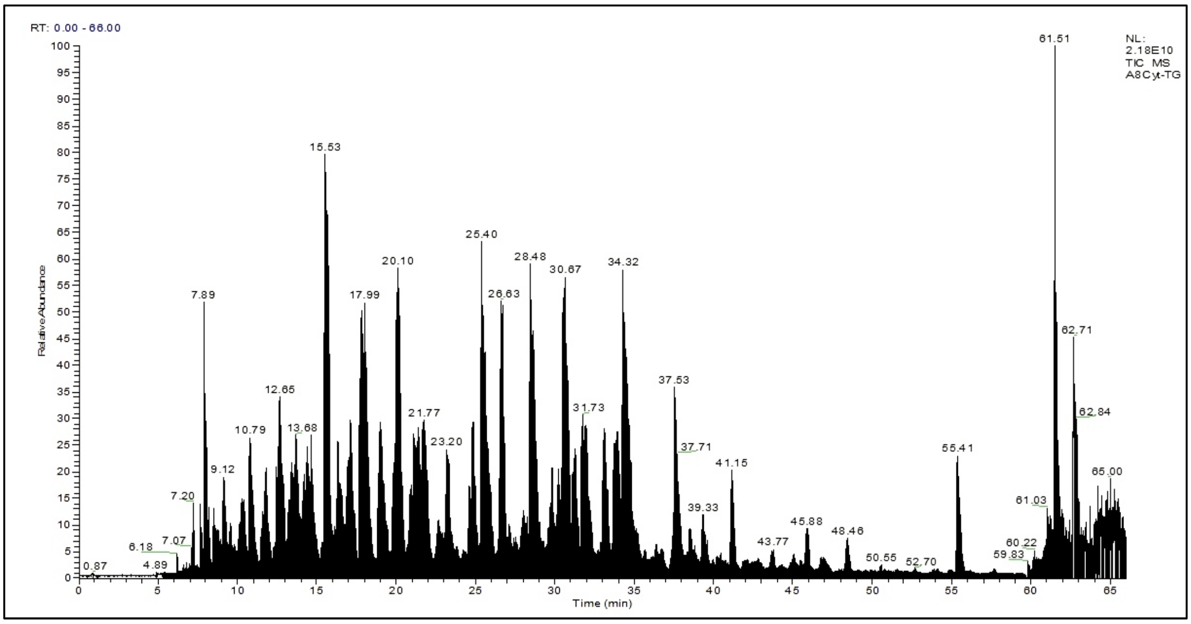


**c.** $\alpha$**8Cyt-Trypsin & Glu-C**

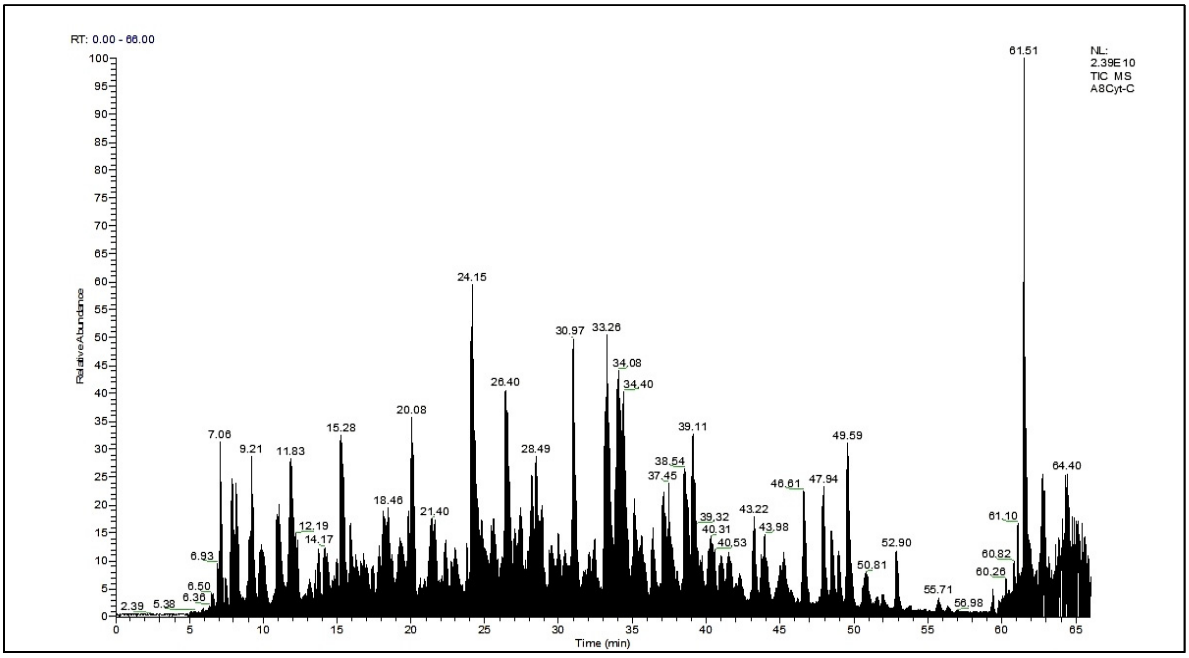

**(B) a. $\alpha$8-Trypsin**

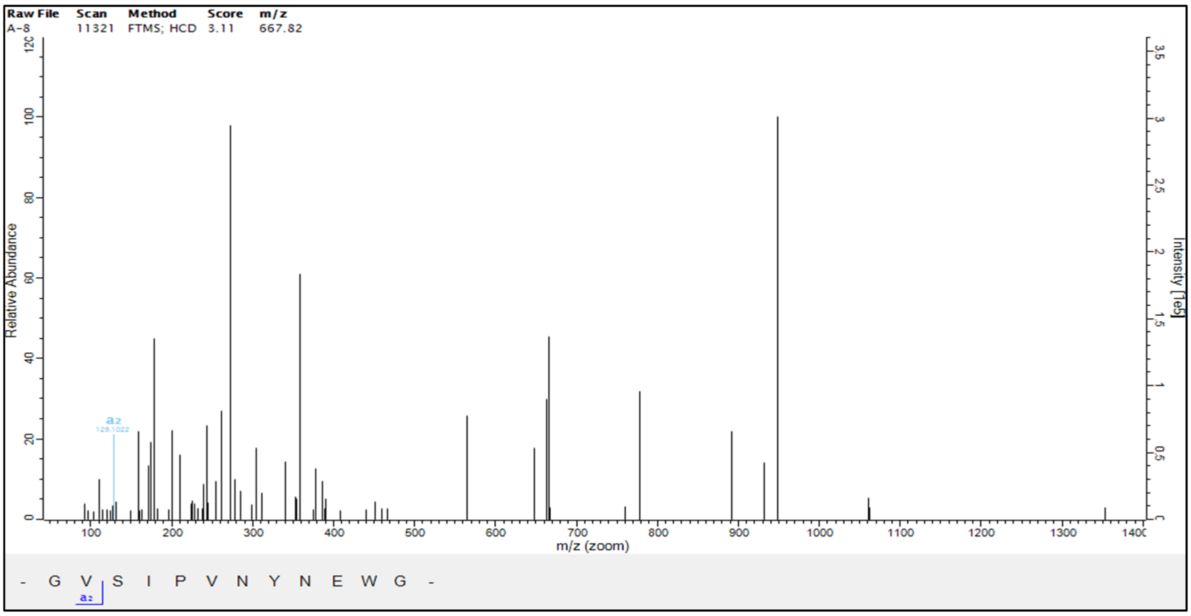


**b. $\alpha$8Cyt-Chymotrypsin**

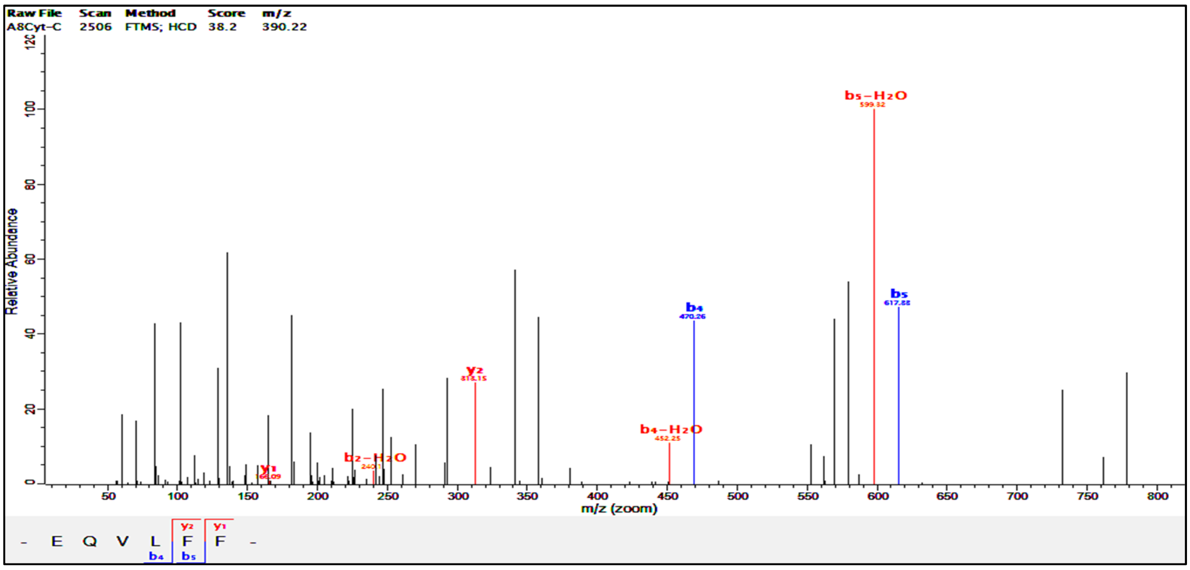

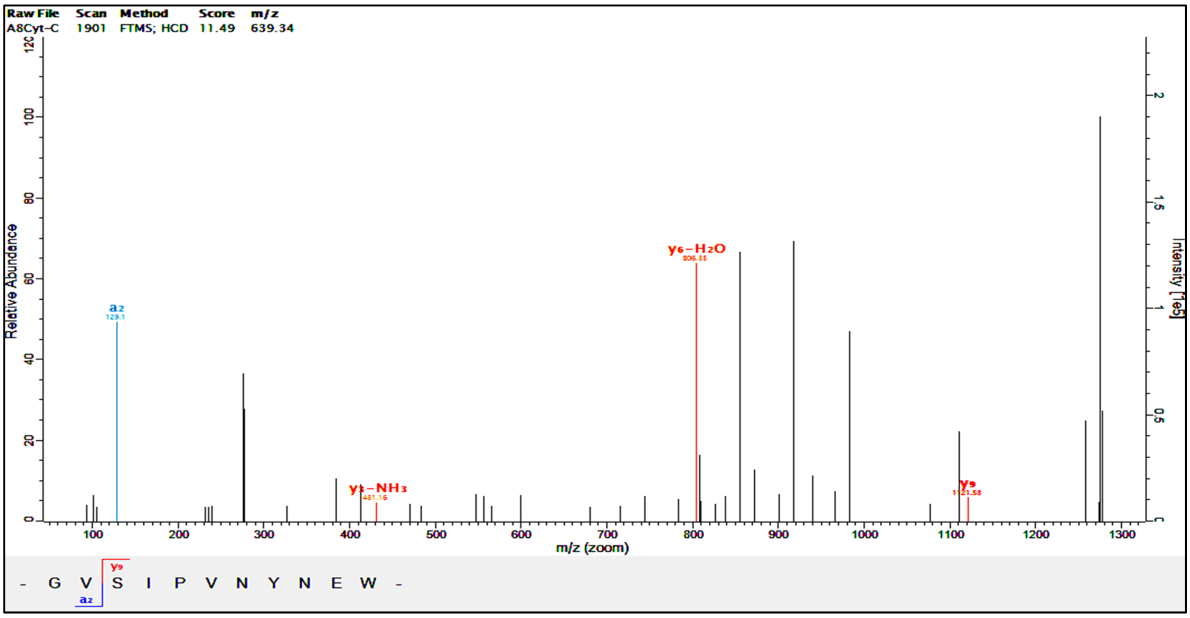
Raw File Scan Method Score m/z
A8Cyt-C 1901 FTMS; HCD 11.49 639.34
Relative Abundance
Intensity [1e5]
m/z (zoom)
a2
y6−H2O
y9
- G V S I P V N Y N E W -

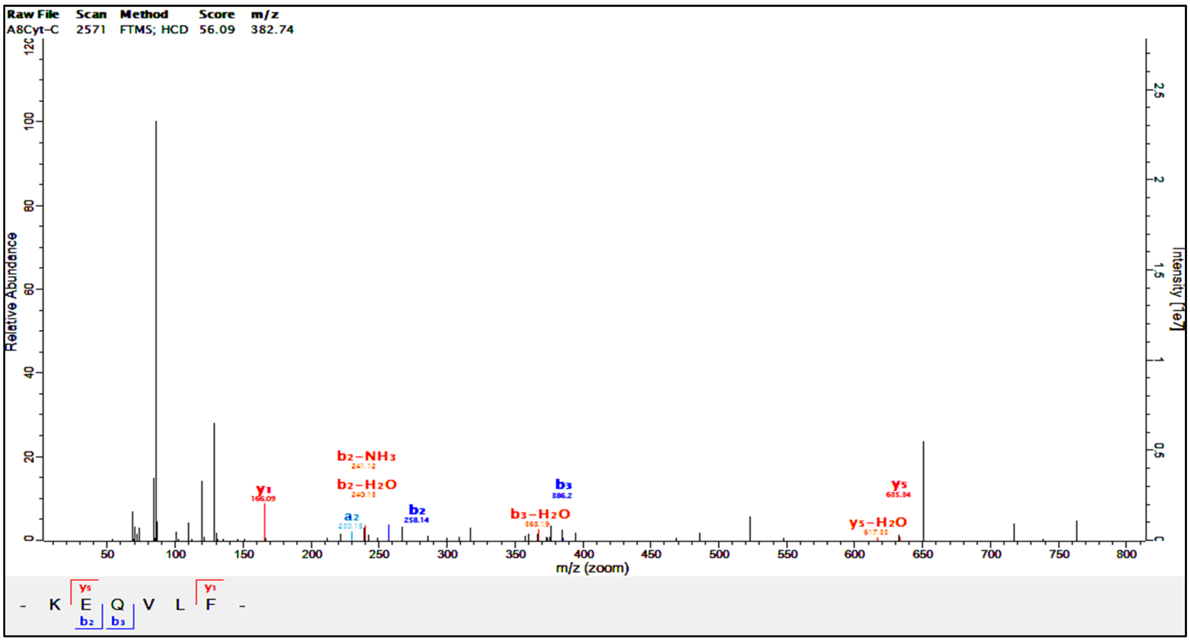
Raw File Scan Method Score m/z
A8Cyt-C 2571 FTMS; HCD 56.09 382.74
Relative Abundance
Intensity [1e7]
m/z (zoom)
y1
b2−NH3
b2−H2O
a2
b2
b3
b3−H2O
y5
y5−H2O
- K E Q V L F -

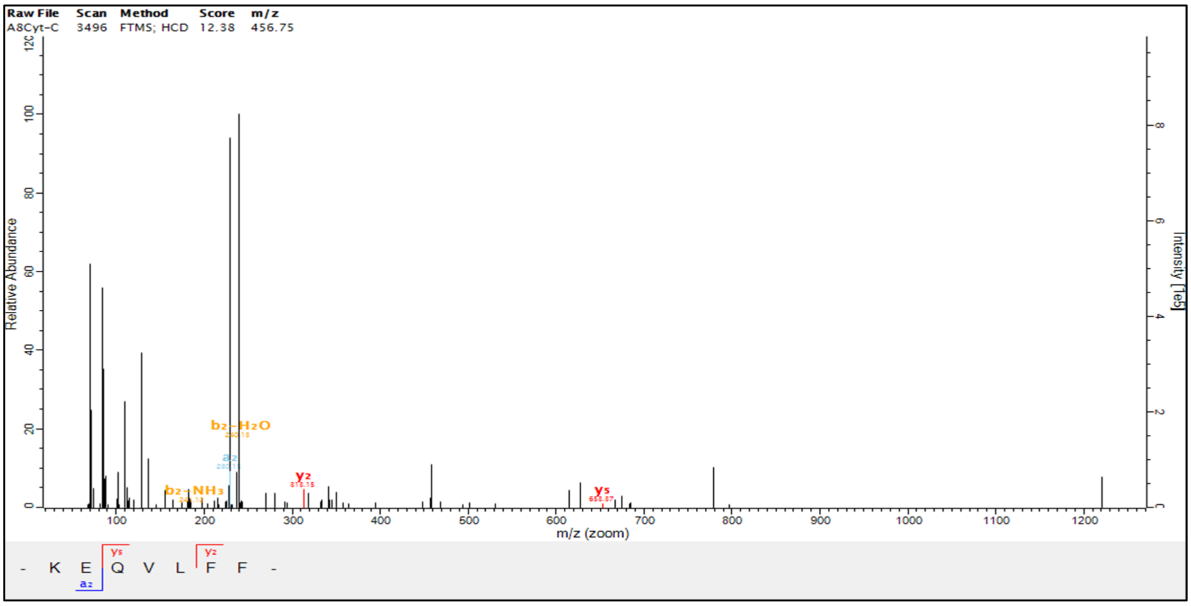
Raw File Scan Method Score m/z
A8Cyt-C 3496 FTMS; HCD 12.38 456.75
Relative Abundance
Intensity [1e5]
m/z (zoom)
b2-H2O
a2
b2-NH3
y2
y5
- K E Q V L F F -

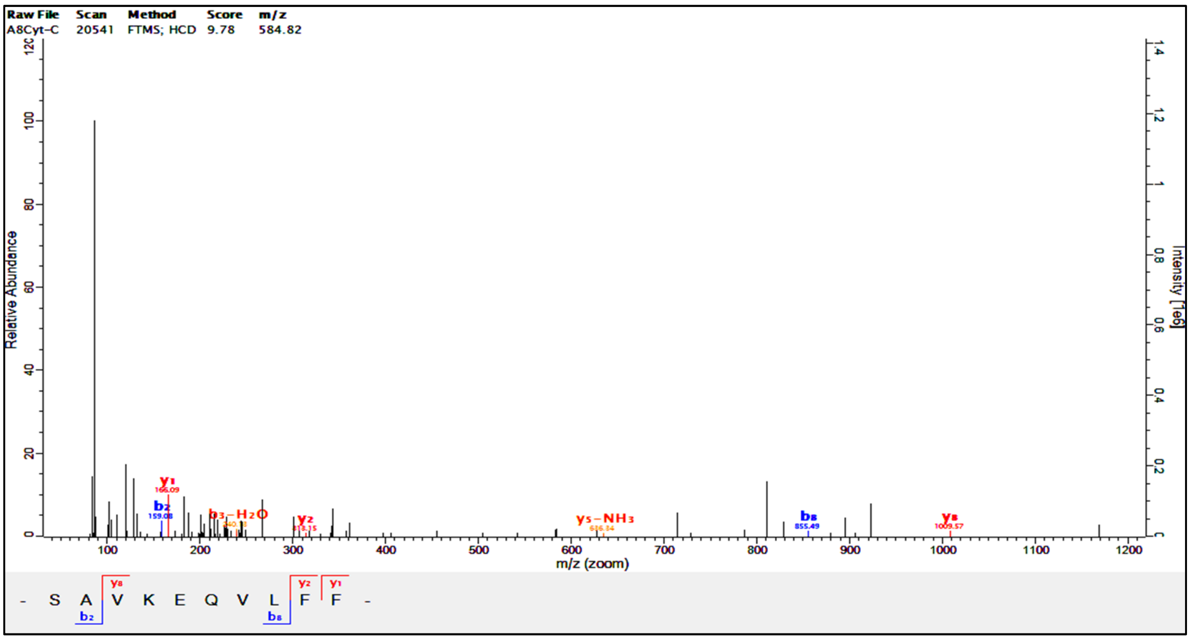
Raw File Scan Method Score m/z
A8Cyt-C 20541 FTMS; HCD 9.78 584.82
Relative Abundance
Intensity [1e6]
m/z (zoom)
y1
b2
b3-H2O
y2
y5-NH3
b8
y8
- S A V K E Q V L F F -

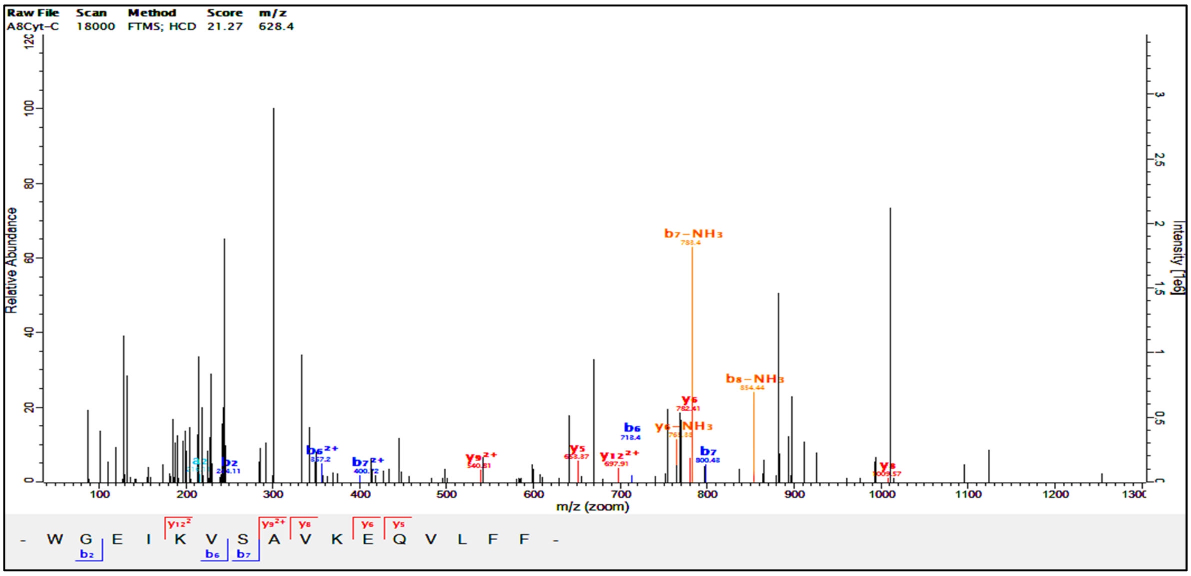


## c. $\alpha$8Cyt-Trypsin & Glu-C

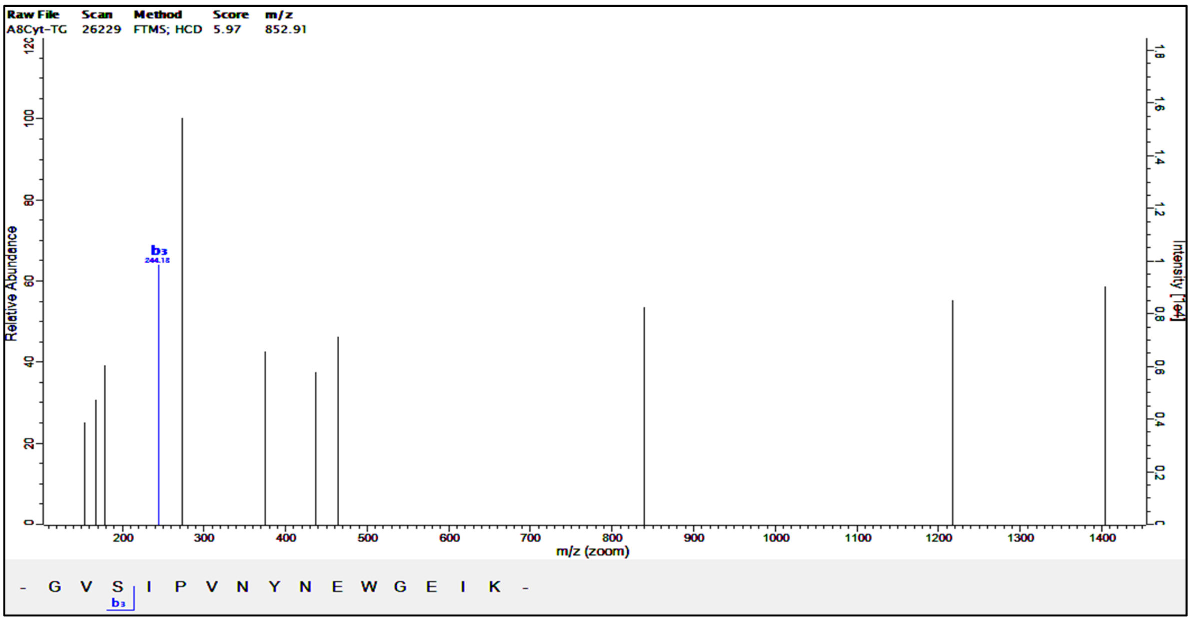

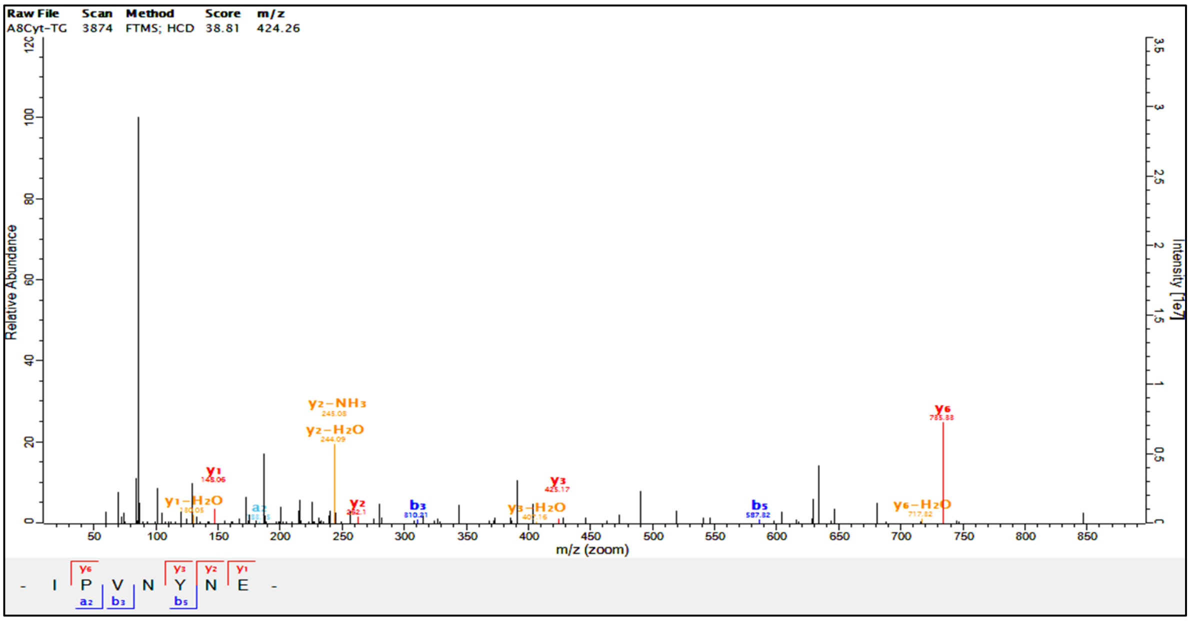

Raw File Scan Method Score m/z
A8Cyt-TG 3874 FTMS; HCD 38.81 424.26
Relative Abundance
Intensity [1e7]
m/z (zoom)
y2−NH3
y2−H2O
y1
y1−H2O
y2
b3
y3
y3−H2O
b5
y6
y6−H2O
- I P V N Y N E -


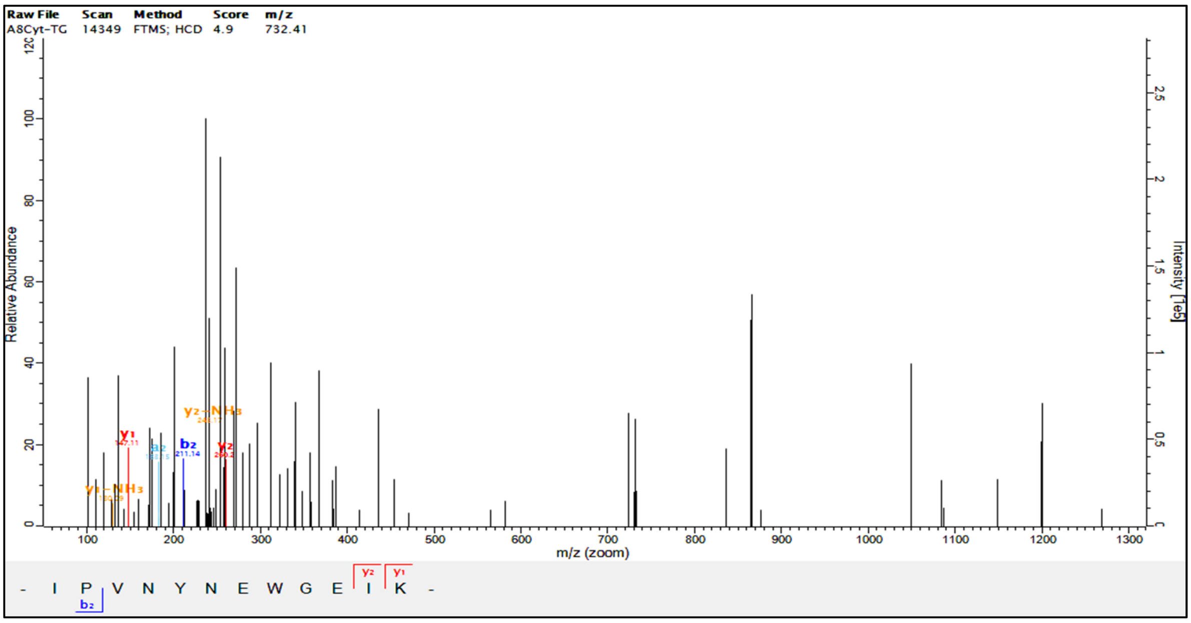

Raw File Scan Method Score m/z
A8Cyt-TG 14349 FTMS; HCD 4.9 732.41
Relative Abundance
Intensity [1e5]
m/z (zoom)
y1
b2
y2
y2−NH3
- I P V N Y N E W G E I K -

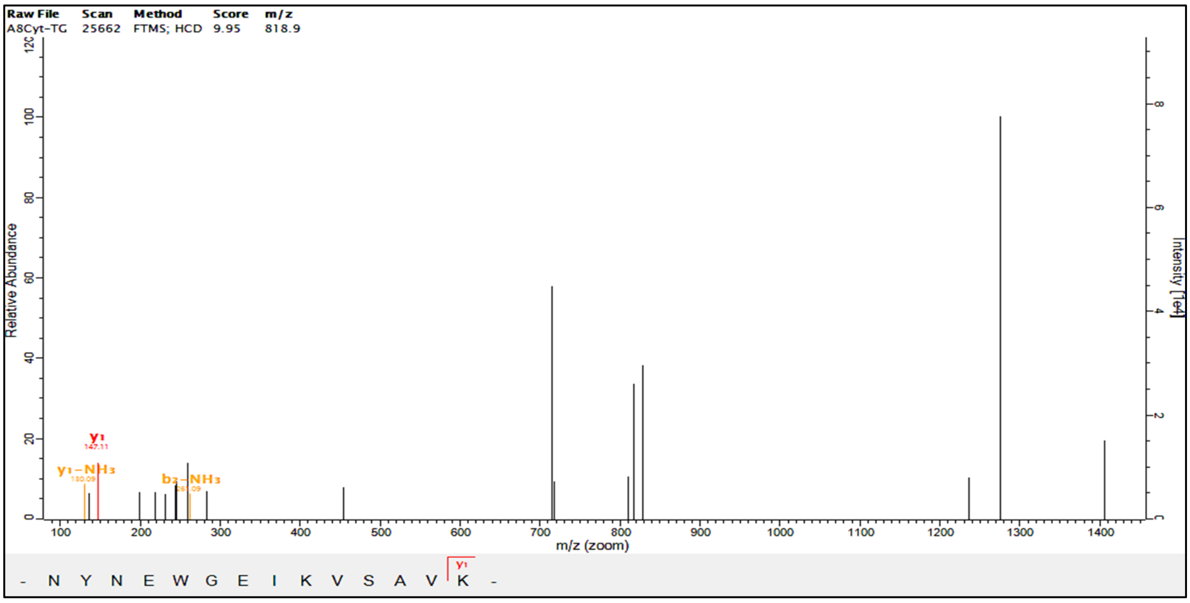
Raw File Scan Method Score m/z
A8Cyt-TG 25662 FTMS; HCD 9.95 818.9
Relative Abundance
Intensity [1e4]
m/z (zoom)
y1
y1−NH3
b2−NH3
- N Y N E W G E I K V S A V K -

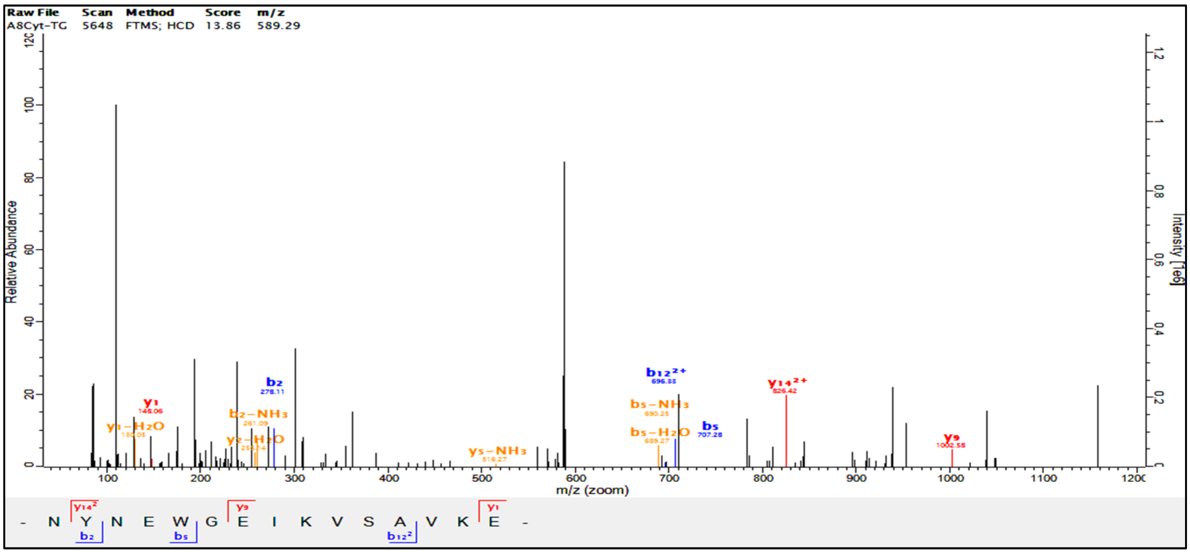
Raw File Scan Method Score m/z
A8Cyt-TG 5648 FTMS; HCD 13.86 589.29
Relative Abundance
Intensity [1e6]
m/z (zoom)
b2
b12²⁺
y14²⁺
b5−NH3
b5−H2O
b5
y9
y1
y1−H2O
b2−NH3
y5−NH3
- N Y N E W G E I K V S A V K E -

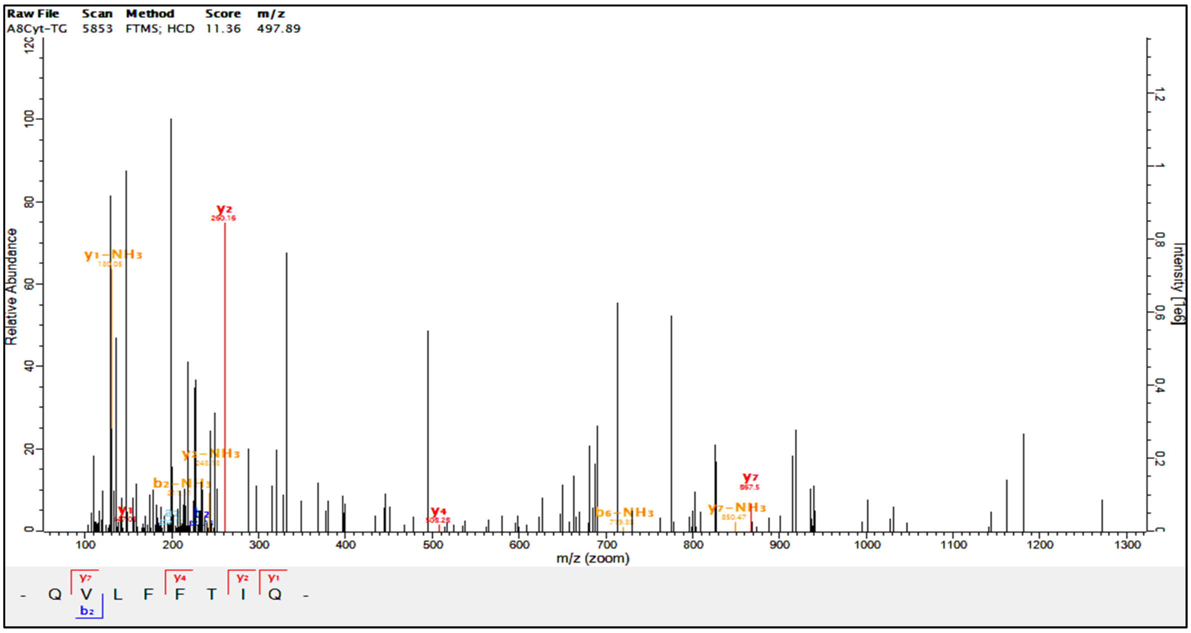
Raw File Scan Method Score m/z
A8Cyt-TG 5853 FTMS; HCD 11.36 497.89
Relative Abundance
Intensity [1e6]
m/z (zoom)
y2
y1−NH3
y7
y4
b6−NH3
y7−NH3
- Q V L F F T I Q -

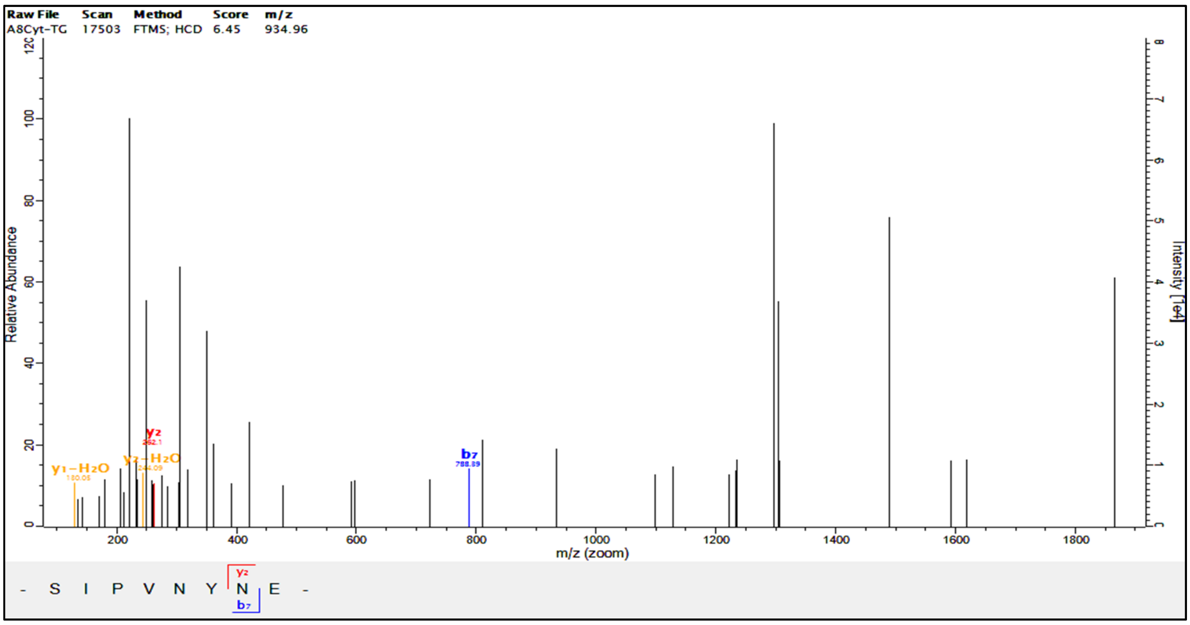
Raw File Scan Method Score m/z
A8Cyt-TG 17503 FTMS; HCD 6.45 934.96
Relative Abundance
Intensity [1e4]
m/z (zoom)
y2
y1−H2O
b7
- S I P V N Y N E -

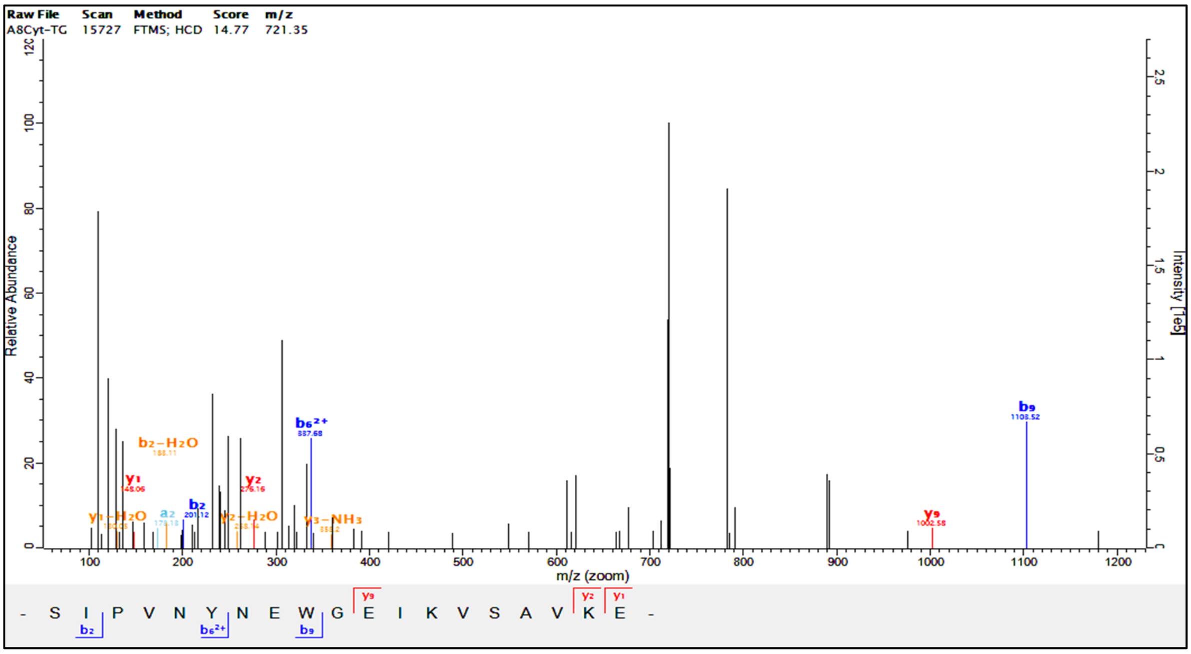

**Figure S2.** Representative total ion chromatograms and MS/MS spectrum of α8-AeDNV and α8Cyt-AeDNV. (A) Total ion chromatograms (sum of all ion strength versus time) of (a) loop α8 and (b,c) loop α8Cyt (B) MS/MS spectrum of each peptide in loop α8 and loop α8Cyt after enzyme digestion. (a) Loop α8 was digested by trypsin while (b, c) loop α8Cyt was digested by chymotrypsin and tripsin&Glu-C.

**Table S1. The raw data collected by mass spectrometry through the MaxQuant database and the obtained results of protein identification.**

| Protein IDs | Unique peptides | Unique Sequence coverage [%] | Mol. weight (kDa) | Sequence length (aa) | Score | Intensity |
|---|---|---|---|---|---|---|
| α8 (trypsin) | 1 | 100 | 1.3344 | 12 | 68.712 | 202590000 |
| α8Cyt-Chymotrypsin | 6 | 89.7 | 3.2957 | 29 | 73.3 | 5.812E+09 |
| α8Cyt-Trypsin& Glu-C | 8 | 100 | 3.2957 | 29 | 113.31 | 3.363E+09 |
